# Cortical Thickness Differences Are Associated With Chemical Synaptic Transmission Upregulated Genes in Degeneration of Mild Cognitive Impairment

**DOI:** 10.3389/fnagi.2021.745381

**Published:** 2021-10-29

**Authors:** Suping Cai, Kexin Huang, Fan Yang, Xuwen Wang, Sijia Wu, Yubo Wang, Liyu Huang

**Affiliations:** School of Life Sciences and Technology, Xidian University, Xi’an, China

**Keywords:** cortical thickness differences, chemical synaptic transmission, partial least squares regression, weighted gene co-expression network analysis, structural magnetic resonance images, cortical gene expression

## Abstract

Mild cognitive impairment (MCI) is a transition between normal cognition (NC) and Alzheimer’s disease (AD). Differences in cortical thickness (ΔCT) have been reported in cases that degenerate from MCI to AD. The aspects of genetic and transcriptional variation related to ΔCT are vague. In this study, using an 8-year longitudinal follow-up outcome, we investigated the genetic correlates of ΔCT in MCI subjects with degeneration from MCI to AD (MCI_AD). We employed partial least squares regression (PLSR) on brain T1-weighted magnetic resonance imaging (MRI) images of 180 participants [143 stable MCI (MCI_S) participants and 37 MCI_AD participants] and brain gene expression data from the Allen Institute for Brain Science (AIBS) database to investigate genes associated with ΔCT. We found that upregulated PLS component 1 ΔCT-related genes were enriched in chemical synaptic transmission. To verify the robustness and specificity of the results, we conducted PLSR analysis invalidation and specificity datasets and performed weighted gene co-expression network analysis instead of PLSR for the above three datasets. We also used gene expression data in the brain prefrontal cortex from the Gene Expression Omnibus (GEO) database to indirectly validate the robustness and specificity of our results. We conclude that transcriptionally upregulated genes involved in chemical synaptic transmission are strongly related to global ΔCT in MCI patients who experience degeneration from MCI to AD.

## Introduction

Mild cognitive impairment (MCI) refers to the prodrome of Alzheimer’s disease (AD) (Stephan et al., 2012). Longitudinal studies have found that some MCI patients maintain an MCI state, and some MCI patients experience degeneration to AD over a follow-up period (Morris and Price, 2001). The potential reason for this difference in MCI conversion is worth exploring. Structural abnormalities in brain morphology, such as a decrease or increase in cortical thickness in particular brain regions, are an important biomarker of MCI or AD (Lerch et al., 2005; Querbes et al., 2009; Filippi et al., 2020). Previous studies have reported that aberrant cortical thickness is a potential biomarker for tracking cognitive and neuropathological symptoms of MCI progression (Julkunen et al., 2009, 2010; Li et al., 2011). However, it is not clear which aspects of variation are related to the differences in cortical morphology in MCI patients.

Complex genetic risk factors are believed to contribute to 70% of AD risk (Lambert et al., 2013). Varying degrees of genetic variation have a significant relationship with the progression of MCI (Lacour et al., 2017). A meta-analysis using blood from MCI and AD patients found that abnormal transcription was related to MCI progression and might be applied to diagnostic and prognostic biomarkers (Bottero and Potashkin, 2019). Several other genetic studies have revealed that the underlying pathophysiology of AD is sophisticated, encompassing up to thirty associated risk genes that are involved in a set of biological processes, such as lipid metabolism and immune system function (Sims et al., 2017; Tasaki et al., 2018; Jansen et al., 2019; Kunkle et al., 2019). Berchtold et al. (2013) revealed that many synaptic genes were upregulated in MCI patients (such as neurotransmitter receptors and synaptic stabilization genes). Their results suggest that a mechanism that rebalances synaptic transmission exists in the brains of MCI patients. In other words, these upregulated genes may be responsible for AD risk or may compensate for upstream biological and cellular changes. The sophistication of AD pathology indicates that patients may need treatment that is tailored with regard to affected biological processes.

Despite crucial progress in comprehending brain cortical morphology and genetic risk for MCI or AD, a question remains: is there an association between differences in cortical morphology and transcriptional gene expression? Recently, our research team applied weighted gene co-expression network analysis (WGCNA) (Zhang and Horvath, 2005; Langfelder and Horvath, 2008) and preliminarily bridged the brain T1-magnetic resonance imaging (MRI) features and transcriptional expression in MCI participants who progressed to AD (Wang et al., 2021). Romero-Garcia et al. (2019) applied partial least squares regression (PLSR) to determine that synaptic and transcriptionally downregulated genes are associated with cortical thickness differences in autism. PLSR is a data reduction technique closely related to principal component analysis (PCA) and ordinary least squares (OLS) regression (Tobias, 1995). One of the PLSR components is calculated to explain the maximum covariance between the dependent and independent variables. Coincidently, the maximum covariance between differences in cortical morphology and transcriptional gene expression might be calculated by PLSR. Several previous studies have used PLSR to explore the relationship between brain features and gene expression in major depressive disorder or healthy adolescence (Romero-Garcia et al., 2020; Li et al., 2021).

**FIGURE 1:**
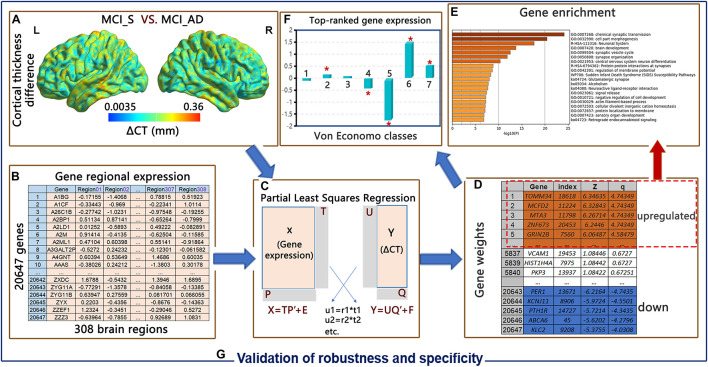
Flowchart of this study. **(A)** Cortical thickness difference (ΔCT) of 308 brain regions between the MCI_S and MCI_AD groups. **(B)** Regional expression of 20,647 genes in 308 brain regions from the AIBS. **(C)** Partial least squares regression (PLSR) analysis using gene expression as a predictor variable and ΔCT as a response variable. **(D)** PLSR weights of all genes in line with its contribution to the overall model in each component (FDR correction, z-score > 1.96). **(E)** Upregulated genes are tested for enrichment analysis and spatial expression of von Economo classes. **(F)** Validation of robustness and specificity of our results. **(G)** Validation of robustness and specificity.

Intuitively, it is possible that risk gene mutations lead to changes in brain structural characteristics, which in turn contribute to changes in clinical phenotypes. At a deeper level, considering the heterogeneity in the results of MCI or AD imaging studies (Shirotani et al., 2017), it is crucial to investigate how the genetic risk is related to the cortical morphological variation observed in MCI patients. Therefore, the purpose of this research was to detect the molecular correlation of disease-related neuroanatomical differences. Here, 180 participants with MCI were followed up for 8 years. Thirty-seven patients with MCI progressed to AD during the 8 years; the rest of the 143 MCI subjects remained in the MCI state. Focusing on cortical thickness differences (ΔCT) from the 180 MCI participants, we asked two key questions: (1) which genes and biological processes are related to ΔCT in degeneration from MCI to AD? and (2) what is the spatial profiling of the genetic expression related to ΔCT? We conducted PLSR and WGCNA analyses and combined the ΔCT measured by T1-MRI in MCI patients with postmortem gene expression data downloaded from the Allen Institute for Brain Science (AIBS) (Hawrylycz et al., 2012, 2015) to address the two questions. We then validated the robustness and specificity of our results with validation datasets. The flow chart of the current research ideas is shown in Figure 1.

## Materials and Methods

### Ethics Statement

The T1-MRI images we evaluated for this work were from the Alzheimer’s Disease Neuroimaging Initiative (ADNI) database.^1^ We confirm that all procedures performed in this study involving human participants were in accordance with the ethical standards of the ADNI consortium Ethics Committee and with the 1964 Helsinki declaration and its later amendments or comparable ethical standards. Written informed consent was obtained from all participants or surrogates (see text footnote 1).

### Discovery Dataset: T1-Magnetic Resonance Imaging Data and Quality Control

We tracked 189 MCI subjects from the ADNI database for 8 years. On the last record (September 2020), 147 MCI subjects were stable in the MCI state (MCI_S), and 42 MCI subjects had progressed to AD (MCI_AD). The timeline of the transition to AD is shown in Supplementary Table 1. T1-weighted magnetization prepared rapid gradient echo (MPRAGE) MRI data at baseline (tracking start time: September 2012) were used. The thickness of the cortex was obtained employing FreeSurfer (Fischl, 2012). The brain gray matter was parcellated into 308 cortical regions, and the mean cortical thickness for the 308 regions was obtained. The detailed quality control and neuroimaging processing are shown in the Supplementary Materials. The final sample consisted of 143 MCI_S and 37 MCI_AD patients matched by sex and age. Details are in Table 1.

**TABLE 1:** Demographic information in discovery dataset, validation dataset 1 and specificity dataset 1.

Dataset	Discovery dataset (ADNI)		Validation dataset 1 (ADNI)		Specificity dataset 1 (ADNI)	
			
Groups	MCI_S^a^	MCI_AD	NC_S^b^	MCI_S^a^	NC_S^b^	NC_MCI
*n*	143	37	53	143	53	30
Sex (M/F)	(80/63)	(26/11)	(21/32)	(80/63)	(21/32)	(16/14)
Age	71.41 ± 7.59	73.35 ± 7.19	75.45 ± 5.96	71.41 ± 7.59	75.45 ± 5.96	77.57 ± 6.24
CDR	0.05 ± 0.00	0.05 ± 0.00	0.00 ± 0.00	0.05 ± 0.00	0.00 ± 0.00	0.05 ± 0.00
MMSE	28.31 ± 1.57	27.57 ± 1.82	29.09 ± 1.06	28.31 ± 1.57	29.09 ± 1.06	28.63 ± 1.847

*Data are the mean ± standard deviation; statistical tests showed no significant differences in sex, age, MMSE or CDR (*p* > 0.01, FDR correction). The n-row represents the number of participants.*

*MCI_S: stable MCI; MCI_AD: transition from MCI to AD; NC_S: remaining normal cognitive state; NC_MCI: conversion from NC to MCI; CDR: clinical dementia rate; MMSE: Mini-Mental State Examination.*

*^*a*^Indicates that the same MCI_S group was used for both the discovery and validation datasets.*

*^*b*^Indicates that the same NC_S group was used for both the validation and specificity datasets.*

Differences in cortical thickness (ΔCT) were calculated as follows. The cortical thickness (CT) of MCI_S has a matrix of 308 × 143, and MCI_AD has a matrix of 308 × 37. For instance, brain region *A* in the MCI-S group has a mean CT_MCI–S_ ((CT_1_ + CT_2_…CT_143_)/143), and in the MCI_AD group, it has a mean CT_MCI_AD_ ((CT_1_ + CT_2_…CT_37_)/37). Then, the ΔCT of brain region *A* is the mean CT_MCI–S_ minus the mean CT_MCI_AD_. As there are 308 cortical regions, the ΔCT has a matrix of 308 × 1.

### Discovery Dataset: Gene Expression Data

The genetic expression dataset was acquired from the AIBS database^2^ (Hawrylycz et al., 2012, 2015). We evaluated T1-MRI images from each AIBS donor to determine the brain structure corresponding to each tissue sample. We used FreeSurfer (fsaverage) to parcellate the brain structure into 308 cortical regions (Fischl, 2012); the brain structure was then warped from the anatomical space into the surface reconstruction of each donor’s brain from the AIBS. Pertinently, 20,647 gene expression values were provided by the AIBS. Thus, a 308 × 20,647 matrix that included the whole genome expression values for the 308 brain regions was obtained after processing (Romero-Garcia et al., 2018). More details are shown in the Supplementary Materials. The codes used to process these data are in this link.^3^ Brain imaging was performed using a BrainNet viewer (Xia et al., 2013).

### Discovery Dataset: Partial Least Squares Regression Analysis

Partial least squares regression was employed to ascertain which genes had a significant association with ΔCT. Then, weighted partial least squares (PLS) values that were z-transformed for each gene were obtained [false discovery rate (FDR) correction, adjusted *p* < 0.05]. We chose genes that passed FDR correction with both positive and negative weights for the next step. The detailed steps of the PLSR analysis and the bases of choosing the PLS components are described in the Supplementary Materials.

### Discovery Dataset: Enrichment Analysis

We used the Metascape tool^4^ (Zhou et al., 2019) to perform enrichment analysis with the Benjamini–Hochberg FDR correction (*q* < 0.05) of the significant genes from selected PLSR components [Gene Ontology (GO) biological processes, Kyoto Encyclopedia of Genes and Genomes (KEGG) pathways, and protein--protein interaction networks]. We performed tissue-specific enrichment analysis using Enrichr^5^ [FDR correction (*q* < 0.05)] (Chen et al., 2013; Kuleshov et al., 2016). Detailed information is shown in the Supplementary Materials.

### Validation and Specificity

To verify the robustness of our results, we utilized the other independent dataset, which was from the ADNI database, as the validation dataset. Two groups, one consisting of 143 MCI_S patients and the other consisting of 53 individuals with normal cognition (NC), served as validation dataset 1. PLSR analysis and enrichment analysis were conducted in validation dataset 1. To verify that our results were MCI specific, we used another independent dataset as the specificity dataset, which was also from the ADNI database. Eighty-three normal cognitive participants were longitudinally followed for 8 years. During the 8-year period, 30 participants transitioned from normal cognitive function to MCI (NC_MCI). The other 53 participants remained in a normal cognitive state (NC_S). We selected the baseline T1-MRI images of 83 normal cognitive participants (53 individuals with NC and 30 NC_MCI participants) as specificity dataset 1. Gene expression data were the same as the discovery dataset. To process T1-weighted MRI images, PLSR analysis and enrichment analysis of the two datasets were conducted with the same pipeline as described above.

### Validation Using the Weighted Gene Co-expression Network Analysis Method

To remove the influence of the method on the results, we performed WGCNA instead of PLSR to verify the reliability of our initial results in the discovery and validation datasets. Detailed processes are provided in the Supplementary Materials.

### Indirect Validation and Specificity Analyses

Tissue-specific enrichment analysis results showed that the prefrontal lobe was the coincident brain tissue in both the validation and discovery datasets. Therefore, we performed validation and specificity analyses purely from the perspective of gene expression data from the prefrontal cortex to indirectly verify our results. Validation dataset 2 comprises microarray data of 56 AD patients and 44 normal controls from the Gene Expression Omnibus (GEO) database. Specificity dataset 2 of 36 vascular dementia (VaD) patients and 44 normal controls was also acquired from the GEO database. Detailed information is shown in the Supplementary Materials.

### Von Economo Classification for Partial Least Squares Component 1

For the selected PLSR component, we also conducted spatial expression profiling of PLSR component 1 across five Von Economo (VE) classifications (Economo et al., 2008) and two additional subtype classifications, including the limbic system and old cortex and insular lobe (Zilles and Amunts, 2012).

### Assigning ΔCT Related Genes to Cell Types and Enrichment Analysis in the Discovery Dataset

We used the secondary data of cellular markers (gene sets corresponding to each cell type) for reference. The secondary data of cellular markers are based on the data from the AIBS database. Gene sets corresponding to each cell type is available data from Li et al. (2021). Cell-specific gene set is from large-scale single-cell studies of the adult human cortex (Seidlitz et al., 2020). Cell types are divided into seven canonical classes: astrocytes, excitatory neurons, inhibitory neurons, endothelial cells, oligodendrocytes, oligodendrocyte precursors (OPs), and microglia. We overlapped the gene set of each cell type with the PLSR component 1 (PLS1) genes. A permutation test was applied to obtain the *p*-value of the overlapping gene number in each cell type (FDR correction, *p* < 0.05). We also performed enrichment analyses in genes involved in each cell type using the Metascape tool [Benjamini–Hochberg FDR correction (*q* < 0.05)] (Zhou et al., 2019).

## Results

### Demographic Characterization and Partial Least Squares Regression Analysis Results

The demographic characterization of the discovery dataset, validation dataset 1 and specificity dataset 1 is shown in Table 1. The demographic information of validation dataset 2 and specificity dataset 2 is shown in Supplementary Table 1.

**FIGURE 2:**
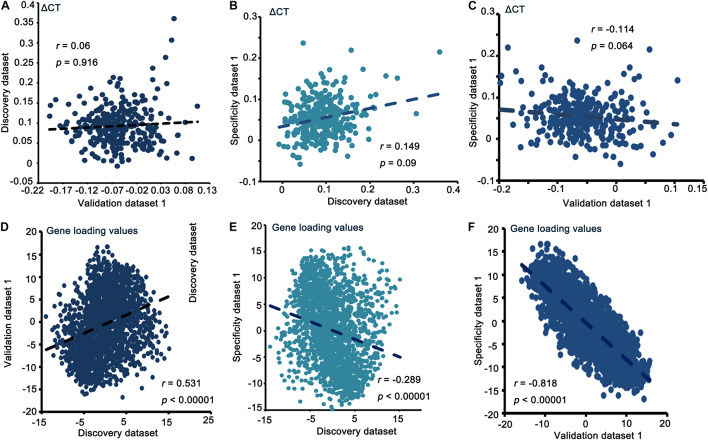
Correlation of ΔCT and gene loading value between the discovery, validation, and specificity datasets **(A–F)**.

In the discovery dataset, we identified that the 16-component model had the best fit from the initial 35 component analyses applying cross-validation. The 16-component model was used in the PLSR analysis. Components 1 and 2 interpreted greater than 10% of the variance. However, only PLS1 interpreted a remarkable ratio of the ΔCT (*p* < 0.001, 10,000 permutations), and it was chosen for subsequent analyses (FDR correction, *q* < 0.05). The correlations of ΔCT and PLS1 gene loading among the discovery dataset, validation dataset 1 and specificity dataset are shown in Figure 2. Detailed PLSR analysis results of these three datasets are described in the Supplementary Materials.

### Gene Enrichment Results in Discovery and Validation Datasets

**FIGURE 3:**
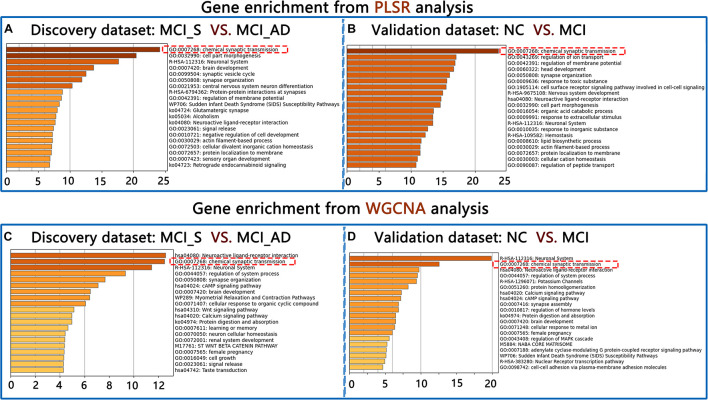
Gene enrichment results from PLSR and WGCNA in the discovery dataset **(A,C)** and the validation dataset **(B,D)**.

In the discovery dataset, the first GO term was “chemical synaptic transmission” (GO: 0007268) in upregulated genes from PLS1 (FDR correction for multiple comparisons [adjusted *p*-value = 0.0005; Log_10_(*q*-value) = −20.072)]. In validation dataset 1, the first GO term was also “chemical synaptic transmission” (GO: 0007268) in upregulated genes. When the WGCNA method was used, the first GO term was also GO: 0007268 in upregulated genes in the discovery and validation datasets. Please see Figure 3. However, no such consistent results were found in downregulated genes.

**FIGURE 4:**
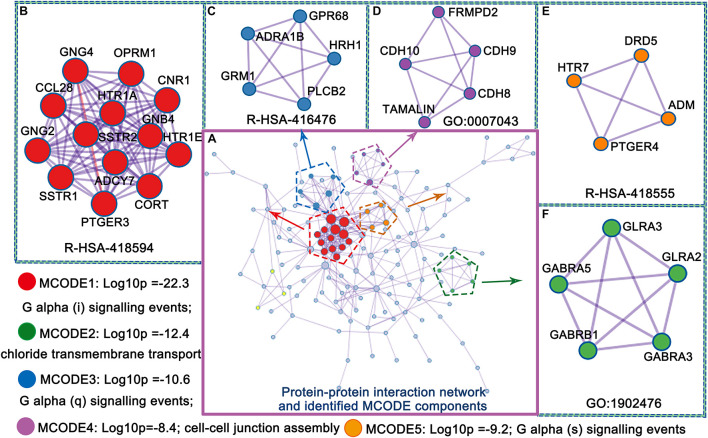
Protein–protein interaction network and identified MCODE components **(A–F)** from PLS1 gene enrichment.

The upregulated genes were mainly involved in the protein–protein interaction network, and the identified molecular complex detection (MCODE) components are shown in Figure 4. Five MCODE components, including G alpha (i,q, and s) signaling events, chloride transmembrane transport and cell-cell junction assembly, were identified. These processes play a key role in MCI progression. We discuss this finding later in more detail.

**FIGURE 5:**
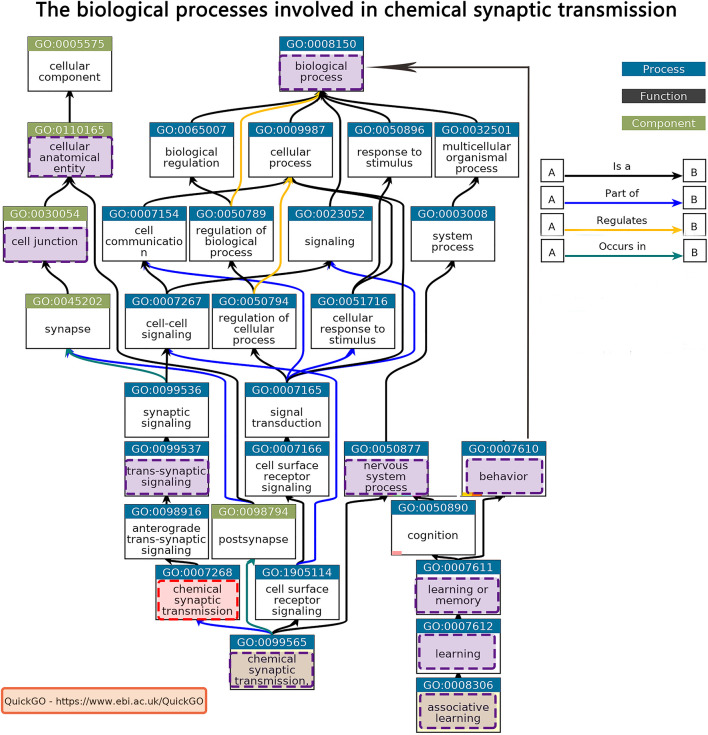
Biological processes involved in chemical synaptic transmission from QuickGO.

Tissue-specific enrichment analysis results showed that the prefrontal lobe was the same brain region of interest in both the discovery and validation datasets (Supplementary Table 8). We used a gene expression dataset of the prefrontal cortex from the GEO to indirectly verify our results. Enrichment analysis results showed that the first GO term was also GO: 0007268 in upregulated genes. Detailed information is provided in the Supplementary Materials. The biological processes involved in GO: 0007268 were extracted by QuickGO^6^ (Figure 5).

### Von Economo Classification Results

**FIGURE 6:**
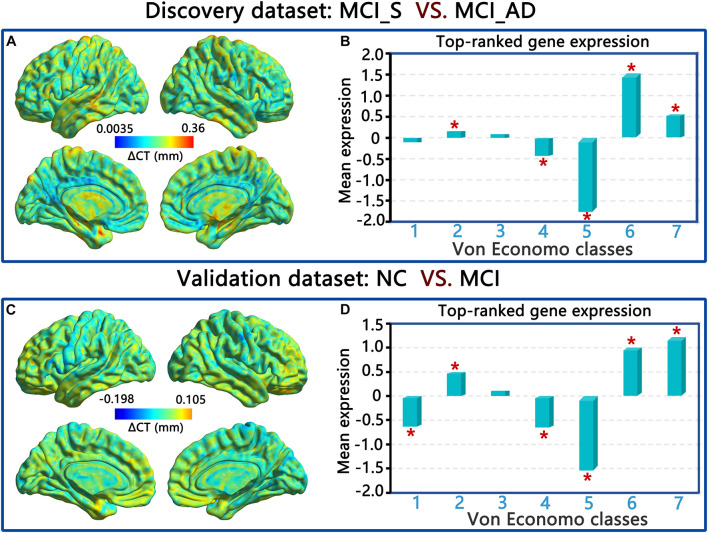
ΔCT and von Economo classes of PLS1 in the discovery dataset **(A,C)**, and the validation dataset **(B,D)**.

In the discovery dataset, PLS1 genes were remarkably overexpressed in the associated cortex, limbic regions, and insular cortex (VE classes 2, 6, and 7) and underexpressed in the secondary sensory cortex and primary sensory cortex (VE classes 4 and 5) compared to a null distribution. In the validation dataset, the genes in PLS1 (also component 1) were also significantly overexpressed in VE classes 2, 6, and 7 and underexpressed in VE classes 1, 4, and 5 compared to a null distribution. The *p*-values for all the above results were corrected by FDR (*p* < 0.01, FDR correction). Please see Figure 6 and the Supplementary Materials.

### Specificity Verification Results

For the PLSR analysis results, we identified 16 components that had the best fit from the initial 35 component analyses. The 16-component model was used in the PLSR analysis. Components 1 and 2 interpreted greater than 10% of the variance. Only PLS1 interpreted a remarkable ratio of the ΔCT (*p* < 0.001, 10,000 permutations). PLS1 was chosen for subsequent analyses (FDR correction, *q* < 0.05). However, the enrichment analysis of PLS1 genes was not enriched in the GO term “chemical synaptic transmission.” For the WGCNA results, enrichment analysis of genes from the most correlated module (MElight green) showed a remarkable association with the GO term “regulation of lipid metabolic process” and was not related to the GO term “chemical synaptic transmission.” This result verifies that the conclusion of our study was specific to MCI.

There were no differentially expressed genes in specificity dataset 2. Less strict thresholds of “*p* < 0.05 (not adjusted) and |log (fold change)| > 1” were applied for the screening of differential mRNA expression. There were almost no differentially expressed genes (two upregulated genes and two downregulated genes) (Supplementary Figure 8). Therefore, we did not carry out enrichment analysis in the next step. This result still verifies that the conclusion of our study was specific to MCI.

### Transcriptional Signatures of ΔCT-Related Genes to Cell Types and Enrichment Analysis Results

**FIGURE 7:**
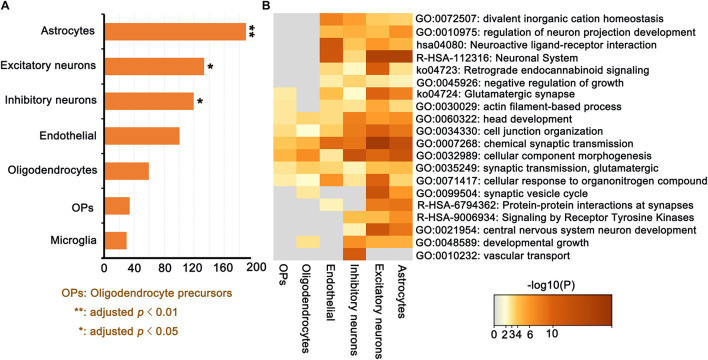
ΔCT-related genes (PLS1) for cell type-specific analysis in discovery dataset. **(A)** The number of overlapping genes for each cell type (all permutated *p*-values were adjusted by FDR; **(B)** Gene Ontology terms enriched for ΔCT related PLS1 genes for each cell type.

A total of 189 genes in the PLS1 gene list were significantly involved in astrocytes (*p* = 0.0096, FDR correction), 133 genes were significantly involved in excitatory neurons (*p* = 0.032, FDR correction), and 119 genes were significantly involved in inhibitory neurons (*p* = 0.041, FDR correction), Furthermore, 100 genes involved in endothelial cells, 59 genes involved in oligodendrocytes, 34 genes involved in OPs, and 30 genes involved in microglia are not significant (Figure 7A). Enrichment analysis results revealed that ΔCT-related genes related to cell types were enriched in GO terms including “chemical synaptic transmission,” “cellular component morphogenesis,” “cell junction organization,” “synaptic transmission, glutamatergic,” and “head development.” Figure 7B shows the detailed information. Together, this study identified ΔCT-related gene expression in unique cell types, allowing us to ascertain specific cell types known to be associated with AD progression pathology.

## Discussion

Applying PLSR to a discovery dataset, we found that PLS1 explained a striking ratio of the ΔCT and that the upregulated genes were remarkably enriched in the GO term “chemical synaptic transmission.” We validated this result using two other independent datasets and the WGCNA method instead of PLSR. Specificity verification was also conducted to verify that the conclusion of our study was MCI specific. Our study provides a stable demonstration coupling disease-related differentiation in cortical thickness with chemical synaptic transmission of transcriptionally upregulated genes from the postmortem brain cortex, thus connecting molecular pathology to macropathology. This might be the first study to combine transcriptionally upregulated gene risk mechanisms in MCI progression with variances in cortical thickness.

The validation using two other independent datasets suggests that our findings are robust. No remarkable correlations of ΔCT between the discovery and validation datasets verified the independence of the validation dataset. We can also verify this from the significant association of gene loading values. Regarding the ΔCT between MCIs with different conversion labels, we could infer that cortical thickness changes during MCI, thus predicting that some MCI patients would become AD patients in the following years. This result is consistent with our previous study (Wang et al., 2021). Abnormal cortical thickness could be attributed to several reasons, for example, alterations in synaptic pruning, myelination and dendritic arborization (Jespersen et al., 2011). The major result of this study is that transcriptionally upregulated genes with chemical synaptic transmission are related to ΔCT in MCI progression.

Alzheimer’s disease is considered a synaptic dysfunction disorder, where toxic oligomers will produce its major effect (Chen et al., 2019). A mouse model study of AD implied that synaptic loss might disturb the excitatory to inhibitory (E/I ratio) balance in pathways vulnerable to AD pathology (Palop et al., 2007). It is expected that chemical synaptic transmission will be an important module in the progression from MCI to AD. In this study, a major finding in the MCODE analysis was the presence of GABA receptor subunits. Some of these genes (e.g., GABRB1) have been found to be upregulated in AD (Limon et al., 2012), which is in agreement with the results from this study. Alterations in GABA receptors which are the main inhibitory neurotransmitter would have large functional implications, such as affecting transmembrane transport function. Similarly, upregulation of glutamatergic synapses was found in this study. Canas et al. (2014) detected a predominant loss of glutamatergic terminal markers in a β-amyloid peptide model of AD. Despite severe glutamate synaptic loss, this result would also have significant implications in excitability and activity dependent production of oligomers (Cirrito et al., 2005; Lauterborn et al., 2021). Moreover, the enrichment analysis of ΔCT related genes to cell types was mainly and significantly enriched in “chemical synaptic transmission” and “synaptic transmission, glutamatergic.” Taken together, this study identified ΔCT related gene expression in unique cell types, allowing us to ascertain specific cell types known to be associated with AD progression pathology. These evidences from transcriptionally upregulated genes in AD or MCI has underlined a crucial function for synaptic transmission for the pathology of this disease (Meng and Mei, 2019; Yan and Rein, 2021). Transcriptional upregulation might imply a causal risk mechanism of AD or a compensatory product of the genetic risk of AD (Scheff et al., 1990).

In this study, we were not able to determine whether transcriptionally upregulated genes causally lead to changes in CT or whether they are both downstream of a common risk mechanism. It is also likely that transcriptional upregulation and changes in CT are downstream processes of the genetic risk involved in MCI progression. It should be noted that upregulated gene transcription is not necessarily responsible for the changes in cortical thickness. However, we were able to demonstrate that changes in CT were significantly associated with chemical synaptic transmission, and this significant association only appeared during the MCI_AD transition and not during the NC_MCI transition (specificity dataset 1). This association did not appear in other subtypes of dementia, such as VaD (specificity dataset 2). Specific analyses in this study verified this result.

In addition to the top GO term “chemical synaptic transmission,” the PLS1 genes were enriched in several GO terms, such as acetylcholine-gated cation-selective channel activity, dopaminergic synaptic transmission, cell junction, cellular anatomical entity, cognition, learning or memory, and behavior. These biological processes are almost inseparable from the functions involved in the etiology of AD or MCI. We summarized the relative GO terms of chemical synaptic transmission from the QuickGO website to explain our enrichment analysis results. Accumulating evidence indicates that abnormal synaptic transmission appears in the MCI stage and is related to deficits in cognition (Querfurth and LaFerla, 2010). Synaptic loss also appears in a variety of transgenic animal models of AD, together with a decline in memory and learning (Sheng et al., 2012; Forner et al., 2017). Nisticò et al. (2012) reported that restoration of excitatory synaptic transmission in the hippocampus through various approaches, such as short peptide gene manipulation, and small molecules or antibodies, could effectively ameliorate cognitive deficits in animal models of AD. Our findings suggest that examining the specific role of synaptic-related genes in changing cortical morphology would facilitate clarifying the underlying molecular mechanisms of ΔCT detected in MCI patients with different conversion labels, creating a new avenue for improving disease-modifying approaches to slow down or treat AD progression. Similarly, the associated pathway of chemical synaptic transmission might be a good starting point.

Enrichment analysis of tissue-specific genes revealed that the genes from PLS1 were enriched in the prefrontal lobe in both the discovery and validation datasets. This indicates that the prefrontal cortex is a key brain region with transcriptional abnormalities. The abnormality might be related to synaptic transmission function. Reddy et al. (2005) reported that compared with individuals with NC, prefrontal synaptic protein loss was more severe than parietal synaptic protein loss in AD participants, implying that the prefrontal regions might be more important for synaptic function. Reddy et al. (2005) also indicated that presynaptic and post-synaptic proteins are critical for synaptic function and might have a relationship with cognitive decline in AD patients. Recently, Yan and Rein (2021) reviewed the pathophysiological implication of the dysregulation of prefrontal synaptic transmission in AD and illustrated the underlying epigenetic mechanisms of the dysregulation of prefrontal genes and synaptic disorders in AD. Our results in AD patients are consistent with these previous findings, which established a remarkable association of cortical thickness and chemical synaptic transmission in the prefrontal lobe.

Protein–protein interaction enrichment analysis showed PLS1 genes mainly involved in G alpha signaling events, chloride transmembrane transport, and cell-cell junction assembly. These pathways have been demonstrated to be related to the pathological process of AD (Georgakopoulos et al., 2001; Thathiah and De Strooper, 2011; Eggert et al., 2018). G-protein-coupled receptors (GPCRs) are the largest class of transmembrane receptors and a common therapeutic target (Dal Prà et al., 2019). These studies provided attractive demonstrations and indicated that GPCRs may have a role in the etiopathogenesis of AD and in many stages of amyloid precursor protein (APP) proteolysis. Deyts et al. (2019) revealed that APP-mediated signaling through a direct interaction with the G-protein alpha subunit prevents memory and cognitive decline in an AD mouse model. Recently, Bose et al. (2021) reported that dysfunction of endosomal and lysosomal CLC chloride transporters results in neurodegeneration in mice and humans. Cell-cell junctions have been shown to be related to the AD-related presenilin 1 gene (Singh et al., 2001). In addition, all three protein functions are targets for FDA-approved small molecule drugs. Again, these previous studies imply the significance of our results.

Spatial profiling showed that PLS1 genes were remarkably overexpressed in the insular cortex, association cortex and limbic regions and underexpressed in the secondary sensory cortex and primary sensory cortex in both the discovery and validation datasets. The association cortex receives multichannel information and connects neural activities in various functional specific areas. A very early study demonstrated that a set of complex visual discrepancies may result from known neuropathology in the visual association cortex in patients with AD (Mendez et al., 1990). Notably, insular cortex atrophy is related to neurological phenotypes in AD (Moon et al., 2014). Recently, a meta-analysis of genetic transcription data emphasized hippocampal synaptic abnormalities in the AD brain (Hosseinian et al., 2020). Similarly, there is evidence that the secondary and primary sensory cortices are closely related to the etiology of AD (Hof and Morrison, 1990).

The limitations of this study cannot be ignored. First, gene transcriptional data were evaluated from only six postmortem adult brain samples, although these types of samples are widely used (Anderson et al., 2018; Romero-Garcia et al., 2019). However, the six adult brain samples were the most spatially detailed gene expression data. Moreover, these data have Montreal Neurological Institute (MNI) coordinates that could map adult brain gene expression to ΔCT. Gene enrichment results related to MCI pathogenesis provide further support for the findings. Second, in this work, we assessed cortical thickness rather than other brain characteristics, such as cortical volume. The combination of the effects of at least two different genetic sources on brain volume would have complicated a significant analysis of the relevant genetic weights. Third, neuronal loss was a factor in the analysis. A very early study showed that neuronal loss correlates with but exceeds neurofibrillary tangles in AD (Gómez-Isla et al., 1997). Nobili et al. (2017) demonstrated that dopamine neuronal loss contributes to memory and reward dysfunction in a model of AD (Nobili et al., 2017). Exploring the associations among neuronal loss, transcriptional level, and abnormal brain cortical morphology is a valuable topic. Fourth, different MCI conversion dates are also a limitation. Finally, the MCI_S group and NC_S group were the same with MCI_S in the validation dataset and NC_S in the specificity dataset (Table 1). The reasons of using the same group is that the MCI_S and NC_S groups are both the control group and to avoid introducing distractions. Romero-Garcia et al. (2019) also used the same control group for both the Autism Discovery and the ADHD datasets. The experimental groups are mutually independent among the three datasets, which is appropriate to validate our findings to a certain degree.

## Data Availability Statement

The datasets presented in this study can be found in online repositories. The names of the repository/repositories and accession number(s) can be found in the article/Supplementary Material.

## Ethics Statement

The studies involving human participants were reviewed and approved by we confirm that all procedures performed in this study involving human participants were in accordance with the ethical standards of the ADNI consortium Ethics Committee and with the 1964 Helsinki declaration and its later amendments or comparable ethical standards. Written informed consent was obtained from all participants or surrogates (adni.loni.usc.edu). The patients/participants provided their written informed consent to participate in this study. Written informed consent was obtained from the individual(s), and minor(s)’ legal guardian/next of kin, for the publication of any potentially identifiable images or data included in this article.

## Author Contributions

SC wrote the first draft of the manuscript. KH and FY prepared the material. XW and SW performed the data acquisition and analysis. YW and LH performed the repeated revisions of the manuscript. All authors contributed to the article and approved the submitted version.

## Conflict of Interest

The authors declare that the research was conducted in the absence of any commercial or financial relationships that could be construed as a potential conflict of interest.

## Publisher’s Note

All claims expressed in this article are solely those of the authors and do not necessarily represent those of their affiliated organizations, or those of the publisher, the editors and the reviewers. Any product that may be evaluated in this article, or claim that may be made by its manufacturer, is not guaranteed or endorsed by the publisher.

## Footnotes

1
adni.loni.usc.edu

2
http://human.brain-map.org

3
https://github.com/SupingCai/PLSR-and-WGCNA-Codes

4
http://metascape.org/

5
http://amp.pharm.mssm.edu/Enrichr

6
http://www.ebi.ac.uk/QuickGO

## References

[B1] Anderson K. M. Krienen F. M. Choi E. Y. Reinen J. M. Yeo B. T. Holmes A. J. (2018). Gene expression links functional networks across cortex and striatum. *Nat. commun.* 9 1–14. 10.1038/s41467-018-03811-x 29651138 PMC5897339

[B2] Berchtold N. C. Coleman P. D. Cribbs D. H. Rogers J. Gillen D. L. Cotman C. W. (2013). Synaptic genes are extensively downregulated across multiple brain regions in normal human aging and Alzheimer’s disease. *Neurobiol. Aging* 34 1653–1661. 10.1016/j.neurobiolaging.2012.11.024 23273601 PMC4022280

[B3] Bose S. He H. Stauber T. (2021). Neurodegeneration upon dysfunction of endosomal/lysosomal CLC chloride transporters. *Front. Cell Dev. Biol.* 9:323. 10.3389/fcell.2021.639231 33708769 PMC7940362

[B4] Bottero V. Potashkin J. A. (2019). Meta-analysis of gene expression changes in the blood of patients with mild cognitive impairment and alzheimer’s disease dementia. *Int. J. Mol. Sci.* 20:5403. 10.3390/ijms20215403 31671574 PMC6862214

[B5] Canas P. M. Simões A. P. Rodrigues R. J. Cunha R. A. (2014). Predominant loss of glutamatergic terminal markers in a β-amyloid peptide model of Alzheimer’s disease. *Neuropharmacology* 76 51–56. 10.1016/j.neuropharm.2013.08.026 24029236

[B6] Chen E. Y. Tan C. M. Kou Y. Duan Q. Ayan A. M. (2013). Enrichr: interactive and collaborative HTML5 gene list enrichment analysis tool. *BMC Bioinform.* 14:128. 10.1186/1471-2105-14-128 23586463 PMC3637064

[B7] Chen Y. Fu A. K. Ip N. Y. (2019). Synaptic dysfunction in Alzheimer’s disease: mechanisms and therapeutic strategies. *Pharmacol. Therap.* 195 186–198. 10.1016/j.pharmthera.2018.11.006 30439458

[B8] Cirrito J. R. Yamada K. A. Finn M. B. Sloviter R. S. Bales K. R. May P. C. (2005). Synaptic activity regulates interstitial fluid amyloid-β levels in vivo. *Neuron* 48 913–922. 10.1016/j.neuron.2005.10.028 16364896

[B9] Dal Prà I. Armato U. Chiarini A. (2019). Family C G-protein-coupled receptors in Alzheimer’s disease and therapeutic implications. *Front. Pharmacol.* 10:1282. 10.3389/fphar.2019.01282 31719824 PMC6826475

[B10] Deyts C. Clutter M. Pierce N. Chakrabarty P. Ladd T. B. Goddi A. (2019). APP-mediated signaling prevents memory decline in Alzheimer’s disease mouse model. *Cell Rep.* 27 1345–1355. 10.1016/j.celrep.2019.03.087 31042463 PMC6508668

[B11] Economo C. V. Koskinas G. N. Triarhou L. C. (2008). *Atlas of Cytoarchitectonics of the Adult Human Cerebral Cortex.* Shenzhen: Kanger International.

[B12] Eggert S. Thomas C. Kins S. Hermey G. (2018). Trafficking in Alzheimer’s disease: modulation of APP transport and processing by the transmembrane proteins LRP1, SorLA, SorCS1c, Sortilin, and Calsyntenin. *Mol. Neurobiol.* 55 5809–5829. 10.1007/s12035-017-0806-x 29079999

[B13] Filippi M. Basaia S. Canu E. Imperiale F. Magnani G. Falautano M. (2020). Changes in functional and structural brain connectome along the Alzheimer’s disease continuum. *Mol. Psychiatry* 25 230–239. 10.1038/s41380-018-0067-8 29743583

[B14] Fischl B. (2012). FreeSurfer. *Neuroimage* 62 774–781. 10.1016/j.neuroimage.2012.01.021 22248573 PMC3685476

[B15] Forner S. Baglietto-Vargas D. Martini A. C. Trujillo-Estrada L. Laferla F. M. (2017). Synaptic impairment in Alzheimer’s disease: a dysregulated symphony. *Trends Neurosci.* 40 347–357. 10.1016/j.tins.2017.04.002 28494972

[B16] Georgakopoulos A. Marambaud P. Robakis N. K. Baki L. (2001). Presenilin−1 is a regulatory component of the cadherin cell adhesion complex: implications for alzheimer’s disease. *Alzheimer’s Dis.* 2001 521–530. 10.1002/0470846453.ch48

[B17] Gómez-Isla T. Hollister R. West H. Mui S. Growdon J. H. Petersen R. C. (1997). Neuronal loss correlates with but exceeds neurofibrillary tangles in Alzheimer’s disease. *Ann. Neurol.* 41 17–24. 10.1002/ana.410410106 9005861

[B18] Hawrylycz M. Miller J. A. Menon V. Feng D. Dolbeare T. Guillozet-Bongaarts A. L. (2015). Canonical genetic signatures of the adult human brain. *Nat. Neurosci.* 18:1832. 10.1038/nn.4171 26571460 PMC4700510

[B19] Hawrylycz M. J. Lein E. S. Guillozet-Bongaarts A. L. Shen E. H. Ng L. Miller J. A. (2012). An anatomically comprehensive atlas of the adult human brain transcriptome. *Nature* 489 391–399. 10.1038/nature11405 22996553 PMC4243026

[B20] Hof P. R. Morrison J. H. (1990). Quantitative analysis of a vulnerable subset of pyramidal neurons in Alzheimer’s disease: II. Primary and secondary visual cortex. *J. Compar. Neurol.* 301 55–64. 10.1002/cne.903010106 1706358

[B21] Hosseinian S. Arefian E. Rakhsh-Khorshid H. Eivani M. Rezayof A. Pezeshk H. (2020). A meta-analysis of gene expression data highlights synaptic dysfunction in the hippocampus of brains with Alzheimer’s disease. *Sci. Rep.* 10 1–9. 10.1038/s41598-020-64452-z 32433480 PMC7239885

[B22] Jansen I. E. Savage J. E. Watanabe K. Bryois J. Williams D. M. Steinberg S. (2019). Genome-wide meta-analysis identifies new loci and functional pathways influencing Alzheimer’s disease risk. *Nat. Genet.* 51 404–413. 10.1038/s41588-018-0311-9 30617256 PMC6836675

[B23] Jespersen S. N. Leigland L. A. Cornea A. Kroenke C. D. (2011). Determination of axonal and dendritic orientation distributions within the developing cerebral cortex by diffusion tensor imaging. *IEEE Trans. Med. Imag.* 31 16–32. 10.1109/TMI.2011.2162099 21768045 PMC3271123

[B24] Julkunen V. Niskanen E. Koikkalainen J. Herukka S.-K. Pihlajamäki M. Hallikainen M. (2010). Differences in cortical thickness in healthy controls, subjects with mild cognitive impairment, and Alzheimer’s disease patients: a longitudinal study. *J. Alzheimer’s Dis.* 21 1141–1151. 10.3233/JAD-2010-100114 21504134

[B25] Julkunen V. Niskanen E. Muehlboeck S. Pihlajamaki M. Kononen M. Hallikainen M. (2009). Cortical thickness analysis to detect progressive mild cognitive impairment: a reference to Alzheimer’s disease. *Dement. Geriat. Cogn. Disord.* 28 389–397. 10.1159/000256274 19907176

[B26] Kuleshov M. V. Jones M. R. Rouillard A. D. Fernandez N. F. Duan Q. Wang Z. (2016). Enrichr: a comprehensive gene set enrichment analysis web server 2016 update. *Nucleic Acids Res.* 2016 W90–W97. 10.1093/nar/gkw377 27141961 PMC4987924

[B27] Kunkle B. W. Grenier-Boley B. Sims R. Bis J. C. Damotte V. Naj A. C. (2019). Genetic meta-analysis of diagnosed Alzheimer’s disease identifies new risk loci and implicates Aβ, tau, immunity and lipid processing. *Nat. Genet.* 51 414–430. 10.1038/s41588-019-0358-2 30820047 PMC6463297

[B28] Lacour E. Louwersheimer H. Hernandez W. Fernandez W. Rosende-Roca Mauleon. (2017). Genome-wide significant risk factors for Alzheimer’s disease: role in progression to dementia due to Alzheimer’s disease among subjects with mild cognitive impairment. *Mol. Psychiatry* 22:153-160 10.1038/mp.2016.18 26976043 PMC5414086

[B29] Lambert J. C. Ibrahimverbaas C. A. Harold D. Naj A. C. Sims R. Bellenguez C. (2013). Meta-analysis of 74,046 individuals identifies 11 new susceptibility loci for Alzheimer’s disease. *Alzheimers Demen.* 9:123. 10.1016/j.jalz.2013.04.040 PMC3896259 24162737

[B30] Langfelder P. Horvath S. (2008). WGCNA: an R package for weighted correlation network analysis. *BMC Bioinform.* 9:1–13. 10.1186/1471-2105-9-559 19114008 PMC2631488

[B31] Lauterborn J. C. Scaduto P. Cox C. D. Schulmann A. Lynch G. Gall C. M. (2021). Increased excitatory to inhibitory synaptic ratio in parietal cortex samples from individuals with Alzheimer’s disease. *Nat. Commun.* 12 1–15. 10.1038/s41467-021-22742-8 33972518 PMC8110554

[B32] Lerch J. P. Pruessner J. C. Zijdenbos A. Hampel H. Teipel S. J. Evans A. C. (2005). Focal decline of cortical thickness in Alzheimer’s disease identified by computational neuroanatomy. *Cereb. Cortex* 15 995–1001. 10.1093/cercor/bhh200 15537673

[B33] Li C. Jian W. Li G. Jian Z. Du H. (2011). Alterations of whole-brain cortical area and thickness in mild cognitive impairment and alzheimer’s disease. *J. Alzheimers Dis.* 27 281–290. 10.3233/JAD-2011-110497 21799248

[B34] Li J. Seidlitz J. Suckling J. Fan F. Ji G.-J. Meng Y. (2021). Cortical structural differences in major depressive disorder correlate with cell type-specific transcriptional signatures. *Nat. Commun.* 12 1–14. 10.1038/s41467-021-21943-5 33712584 PMC7955076

[B35] Limon A. Reyes-Ruiz J. M. Miledi R. (2012). Loss of functional GABAA receptors in the Alzheimer diseased brain. *Proc. Nat. Acad. Sci.* 109 10071–10076. 10.1073/pnas.1204606109 22691495 PMC3382476

[B36] Mendez M. F. Mendez M. A. Martin R. Smyth K. A. Whitehouse P. J. (1990). Complex visual disturbances in Alzheimer’s disease. *Neurology* 40 439–439. 10.1212/WNL.40.3_Part_1.439 2314585

[B37] Meng G. Mei H. (2019). Transcriptional dysregulation study reveals a core network involving the progression of Alzheimer’s disease. *Front. Aging Neurosci.* 11:101. 10.3389/fnagi.2019.00101 31133844 PMC6513962

[B38] Moon Y. Moon W.-J. Kim H. Han S.-H. (2014). Regional atrophy of the insular cortex is associated with neuropsychiatric symptoms in Alzheimer’s disease patients. *Eur. Neurol.* 71 223–229. 10.1159/000356343 24480815

[B39] Morris J. C. Price J. L. (2001). Pathologic correlates of nondemented aging, mild cognitive impairment, and early-stage Alzheimer’s disease. *J. Mol. Neurosci.* 17:101. 10.1385/JMN:17:2:101 11816784

[B40] Nisticò R. Pignatelli M. Piccinin S. Mercuri N. B. Collingridge G. (2012). Targeting synaptic dysfunction in Alzheimer’s disease therapy. *Mol. Neurobiol.* 46 572–587. 10.1007/s12035-012-8324-3 22914888

[B41] Nobili A. Latagliata E. C. Viscomi M. T. Cavallucci V. Cutuli D. Giacovazzo G. (2017). Dopamine neuronal loss contributes to memory and reward dysfunction in a model of Alzheimer’s disease. *Nat. Commun.* 8 1–14. 10.1038/ncomms14727 28367951 PMC5382255

[B42] Palop J. J. Chin J. Roberson E. D. Wang J. Thwin M. T. Bien-Ly N. (2007). Aberrant excitatory neuronal activity and compensatory remodeling of inhibitory hippocampal circuits in mouse models of Alzheimer’s disease. *Neuron* 55 697–711. 10.1016/j.neuron.2007.07.025 17785178 PMC8055171

[B43] Querbes O. Aubry F. Pariente J. Lotterie J.-A. Démonet J.-F. Duret V. (2009). Early diagnosis of Alzheimer’s disease using cortical thickness: impact of cognitive reserve. *Brain* 132 2036–2047. 10.1093/brain/awp105 19439419 PMC2714060

[B44] Querfurth H. LaFerla F. (2010). Alzheimer’s disease. *N. Engl. J. Med.* 362 1844–1845. 10.1056/NEJMra0909142 20107219

[B45] Reddy P. H. Mani G. Park B. S. Jacques J. Murdoch G. Whetsell Jr. (2005). Differential loss of synaptic proteins in Alzheimer’s disease: implications for synaptic dysfunction. *J. Alzheimer’s Dis.* 7 103–117. 10.3233/JAD-2005-7203 15851848

[B46] Romero-Garcia R. Seidlitz J. Whitaker K. J. Morgan S. E. Fonagy P. Dolan R. J. (2020). Schizotypy-related magnetization of cortex in healthy adolescence is colocated with expression of schizophrenia-related genes. *Biol. Psychiatry* 88 248–259. 10.1016/j.biopsych.2019.12.005 32029217 PMC7369635

[B47] Romero-Garcia R. Warrier V. Bullmore E. T. Baron-Cohen S. Bethlehem R. A. (2019). Synaptic and transcriptionally downregulated genes are associated with cortical thickness differences in autism. *Mol. Psychiatry* 24 1053–1064. 10.1038/s41380-018-0023-7 29483624 PMC6755982

[B48] Romero-Garcia R. Whitaker K. J. Seidlitz J. Shinn M. Fonagy P. Dolan R. J. (2018). Structural covariance networks are coupled to expression of genes enriched in supragranular layers of the human cortex. *Neuroimage* 2018:844. 10.1016/j.neuroimage.2017.12.060 29274746 PMC5883331

[B49] Scheff S. W. Dekosky S. T. Price D. A. (1990). Quantitative assessment of cortical synaptic density in Alzheimer’s disease. *Neurobiol. Aging* 11 29–37. 10.1016/0197-4580(90)90059-9 2325814

[B50] Seidlitz J. Nadig A. Liu S. Bethlehem R. A. Vertes P. E. Morgan S. E. (2020). Transcriptomic and cellular decoding of regional brain vulnerability to neurogenetic disorders. *Nat. Commun.* 11 1–14. 10.1038/s41467-020-17051-5 32620757 PMC7335069

[B51] Sheng M. Sabatini B. L. Südhof T. C. (2012). Synapses and Alzheimer’s disease. *Cold Spr. Harbor Persp. Biol.* 4:a005777. 10.1101/cshperspect.a005777 22491782 PMC3331702

[B52] Shirotani K. Asai M. Iwata N. (2017). Paradigm shift from diagnosing patients based on common symptoms to categorizing patients into subtypes with different pathogenic mechanisms to guide treatment for Alzheimer’s disease. *J. Biochem.* 161 463–470. 10.1093/jb/mvx015 28338847

[B53] Sims R. Van Der Lee S. J. Naj A. C. Bellenguez C. Badarinarayan N. Jakobsdottir J. (2017). Rare coding variants in PLCG2, ABI3, and TREM2 implicate microglial-mediated innate immunity in Alzheimer’s disease. *Nat. Genet.* 49 1373–1384. 10.1038/ng.3916 28714976 PMC5669039

[B54] Singh N. Talalayeva Y. Tsiper M. Romanov V. Dranovsky A. Colflesh D. (2001). The role of Alzheimer’s disease-related presenilin 1 in intercellular adhesion. *Experim. Cell Res.* 263 1–13. 10.1006/excr.2000.5098 11161700

[B55] Stephan B. C. M. Hunter S. Harris D. Llewellyn D. J. Siervo M. Matthews F. E. (2012). The neuropathological profile of mild cognitive impairment (MCI): a systematic review. *Mol. Psychiatry* 17 1056–1076. 10.1038/mp.2011.147 22143004

[B56] Tasaki S. Gaiteri C. Mostafavi S. De Jager P. L. Bennett D. A. (2018). The molecular and neuropathological consequences of genetic risk for Alzheimer’s dementia. *Front. Neurosci.* 12:699. 10.3389/fnins.2018.00699 PMC6187226 30349450

[B57] Thathiah A. De Strooper B. (2011). The role of G protein-coupled receptors in the pathology of Alzheimer’s disease. *Nat. Rev. Neurosci.* 12 73–87. 10.1038/nrn2977 21248787

[B58] Tobias R. D. (1995). “An introduction to partial least squares regression,” in *Proceedings of the twentieth annual SAS users group international conference: Citeseer*, (Cary, NC: SAS Institute Inc).

[B59] Wang X. Huang K. Yang F. Chen D. Cai S. Huang L. (2021). Association between structural brain features and gene expression by weighted gene co-expression network analysis in conversion from MCI to AD. *Behav. Brain Res.* 2021:113330. 10.1016/j.bbr.2021.113330 33940051

[B60] Xia M. Wang J. He Y. (2013). BrainNet Viewer: a network visualization tool for human brain connectomics. *PloS One* 8:e68910. 10.1371/journal.pone.0068910 23861951 PMC3701683

[B61] Yan Z. Rein B. (2021). Mechanisms of synaptic transmission dysregulation in the prefrontal cortex: pathophysiological implications. *Mol. Psychiatry* 2021 1–21. 10.1038/s41380-021-01092-3 33875802 PMC8523584

[B62] Zhang B. Horvath S. (2005). A general framework for weighted gene co-expression network analysis. *Stat. Appl. Genet. Mol. Biol.* 4:1128. 10.2202/1544-6115.1128 16646834

[B63] Zhou Y. Zhou B. Pache L. Chang M. Khodabakhshi A. H. Tanaseichuk O. (2019). Metascape provides a biologist-oriented resource for the analysis of systems-level datasets. *Nat. Commun.* 10 1–10. 30944313 10.1038/s41467-019-09234-6 PMC6447622

[B64] Zilles K. Amunts K. (2012). Segregation and wiring in the brain. *Science* 335:1582. 10.1126/science.1221366 22461598

