# Supplementary material for: Cortical Thickness Differences Are Associated With Chemical Synaptic Transmission Upregulated Genes in Degeneration of Mild Cognitive Impairment
Source: Front Aging Neurosci. 2021 Oct 29;13:745381. doi: 10.3389/fnagi.2021.745381 (PMC8585991; doi:10.3389/fnagi.2021.745381)
Supplement: Supplementary file 1 [file Data_Sheet_1.docx]

Cortical thickness differences are associated with chemical synaptic transmission upregulated genes in degeneration of mild cognitive impairment

Suping Cai^#^ (Ph.D.), Kexin Huang^#^ (Ph.D.), FanYang (M.S.), Xuwen Wang (M.S.), Sijia Wu (Ph.D.), Yubo Wang (Ph.D.), Liyu Huang* (Ph.D.)

*School of Life Sciences and Technology, Xidian University, Xi’an, Shaanxi 710071, PR China;*

Supplementary catalogue

1. Neuroimaging Data 2

Discovery dataset 2

Validation Dataset 1 2

Specificity Dataset 1 3

2. Gene Expression Data 3

Discovery Dataset 3

Validation Dataset 2 3

Specificity Dataset 2 3

Table S1: Demographic information in validation dataset 2 and specificity dataset 2 4

3. PLSR analysis 4

Overview of PLSR analysis 4

Discovery dataset 6

Table S2: The relative information of the full 35-component model 6

Figure S1: Variance in X (gene expression) explained by PLS components 7

Table S3: The main enrichment result for PLSR1 in Discovery dataset (MCI_S VS. MCI-AD) 7

Validation dataset 1 8

Figure S2: Variance in X (gene expression) explained by PLS components 8

Table S4: The main enrichment result for PLSR1 in validation dataset (NC VS. MCI_S) 8

Specificity dataset 1 9

Figure S3: Variance in X (gene expression) explained by PLS components 9

Figure S4: Enrichment analysis result of Specificity dataset 1. 9

4. WGCNA Analysis 10

Discovery dataset and validation dataset 1 10

Figure S5: co-expressed gene modules were divided into different modules with different colours. 10

Figure S6: Module-trait (ΔCT) relationship in NC VS. MCI_S and MCI_S VS.MCI_AD. 10

Table S5: The main enrichment result using WGCNA in discovery and validation datasets 11

Specificity dataset 1 11

Figure S7: Module-trait (ΔCT) relationship in discovery dataset, validation dataset 1 and specificity dataset 1. 11

Figure S8: Enrichment analysis result in specificity dataset 1. 12

5. Indirect Validation and Specificity Analysis 12

Table S6: Tissue-specific enrichment analysis 12

Validation dataset 2 (GEO) 12

Figure S9: Differential expression analysis and enrichment analysis results 13

Table S7: The main enrichment result in validation dataset 2 (GEO, NC VS. AD) 13

Specificity dataset 2 (GEO) 14

Figure S10: Differential expression analysis in sample of VaD and normal controls 14

6. KEGG based pathway enrichment 14

Table S8: Top 2-10 pathways enrichment for the discovery and validation datasets using PLSR and WGCNA respectively. 14

7. Enrichment analysis in DisGeNET 15

Figure S11: Enrichment analysis result in DisGeNET 16

8. Protein-protein interaction enrichment analysis 16

9. Von Economo classification 16

References 17

## Neuroimaging Data

### Discovery dataset

Structural T1-weighted MPRAGE images were collected from the Alzheimer’s Disease Neuroimaging Initiative (ADNI) database (adni.loni.usc.edu). From ADNI, we selected a subset of 2 diagnostic groups (MCI_S: stable in MCI state in eight years; MCI_AD: degeneration from MCI to AD in eight years). The initial sample consisted of 189 eligible participants. The ADNI database provided information regarding the motion in a raw anatomical image based on visual inspection. We also used automated Brain Images Database Structure (BIDS) apps to assess raw anatomical MRI scans for quality and output quantitative measurements ([Gorgolewski et al., 2017](#_ENREF_6)). Data quality was also assessed after processing images to ensure that the selected images were truly high quality. Then, the structural data were pre-processed using Freesurfer *v5.3.0* to estimate regional cortical thickness ([Fischl, 2012](#_ENREF_5)). The cortical thickness (CT) maps were automatically parcellated into 308 equally sized cortical regions of 500 mm^2^ that were constrained by the anatomical boundaries defined in the Desikan-Killiany atlas ([Desikan et al., 2006](#_ENREF_3); [Romero-Garcia et al., 2012](#_ENREF_12)). Individual parcellation templates were created by warping this standard template containing 308 cortical regions to each individual image in native space. Lastly, average cortical thickness was extracted for each of the 308 cortical regions in each participant.

### Validation Dataset 1

In order to validate our findings, we used the other independent dataset as the validation dataset which was also from the ADNI database. We selected a subset of 2 diagnostic groups (MCI_S group and control group of NC). Structural T1-weighted images were pre-processed with the same pipeline as described above. Participants that had an overall variance in cortical thickness that was more than 3 standard deviations from the group mean were removed from further analysis. The final sample for validation consisted of 196 participants (143 MCI_S and 53 NC).

### Specificity Dataset 1

In order to verify our results were MCI specific, we used another independent dataset as the specificity dataset which was also from the ADNI database. 83 normal cognitive participants were longitudinally followed for eight years. During the eight years, 30 participants have a transition from normal cognitive to MCI (NC_MCI). The remaining 53 remained normal cognitive state (NC_S). We selected the 2 subsets (53 NCs and 30 NC_MCIs). Structural T1-weighted images were pre-processed with the same pipeline as described above.

## 2. Gene Expression Data

### Discovery Dataset

A gene expression dataset of the adult human brain created by the Allen Institute for Brain Science (AIBS; http://human.brain-map.org) ([Hawrylycz et al., 2015](#_ENREF_7); [Hawrylycz et al., 2012](#_ENREF_8)) was used to determine the expression profile of each brain cortical region. This dataset includes samples from post-mortem brain of six donors (3 Caucasian, 2 African-American, 1 Hispanic) aged 24-57 years. The limited sample size (n=6) and the large variability in age, gender and ethnicity may have a deep impact in the regional transcriptional pattern. In order to address the potential inter-individual differences of gene expression we tested the effect of donor selection using a leave-one-out approach. We found that the six resulting gene expression profiles were highly similar, showing a relative difference. The consistency of gene expression across donors confirms that results reported in present study are not driven by a single donor. This gene expression dataset of AIBS was applied in many previous studies, such as ([Anderson et al., 2018](#_ENREF_1); [Romero-Garcia et al., 2019](#_ENREF_13)).

### Validation Dataset 2

The validation dataset 2 is an indirect validation dataset without T1-MRI data. It is a microarray gene expression data of the brain prefrontal cortex of AD and normal controls. It is from GEO database (http://www.ncbi.nih.gov/geo, GSE122063). The raw data were downloaded as MINiML files. The extracted data were normalized and processed by log_2_ transformation. The microarray data were normalized using the preprocessCore package in R software (version 3.4.1). Probes were converted to gene symbols according to the platform annotation information of the normalized data. Probes with more than one gene were eliminated and the average value was calculated for genes corresponding to more than one probe. As an initial quality control step using variance stabilized counts with individual horse effect removed using the removeBatchEffect function of limma R package. The final sample consisted of 100 participants (56 AD and 44 normal controls).

### Specificity Dataset 2

The specificity dataset 2 is an indirect specificity dataset without T1-MRI data. It is a microarray data of vascular dementia (VaD) and normal controls (NC) (gene expression data from the brain prefrontal cortex). VaD is one of the subtypes of dementia. Thus, we chose VaD to test the specificity of our results. The specificity dataset 2 is also from GEO database (http://www.ncbi.nih.gov/geo, GSE122063). Specificity dataset 2 was processed with the same pipeline as described in the validation dataset 2. The final sample consisted of 80 participants (36 AD and 44 normal controls). Demographic information in validation dataset 2 and specificity dataset 2 is in Table S1.

##### Table S1: Demographic information of validation dataset 2 and specificity dataset 2

| Datasets | Validation dataset 2 (GEO) | | Specificity dataset 2 (GEO) | |
| --- | --- | --- | --- | --- |
| Groups | NC | AD | NC | VaD |
| n | 44 | 56 | 44 | 36 |
| Sex (M / F) | (20/24) | (12/44) | (20/24) | (20/16) |
| Age | 78.18±5.47 | 81.00±6.56 | 78.18±5.47 | 80.88±6.15 |

##

## 3. PLSR analysis

### Overview of PLSR analysis

***The following overview of PLSR and the difference between the SIMPLS and the NIPALS algorithm is from Rafael Romero-Garci et al., 2019.***

Partial least squares regression or PLSR is a data reduction technique closely related to principal component analysis (PCA) and ordinary least squares (OLS) regression. Here we use the SIMPLS algorithm (de Jong, 1993), where the independent variable matrix (X) and the dependent variable (Y) is centred giving rise to X_0_ and Y_0_ respectively. The first component is then weighted by w_1_ and q_1_ to calculate factor scores (or component scores) T_1_ and U_1_.

This T1 is the weighted sum of the centred independent variable:

*T_1_ = X_0_w_1_ + E_1_* (equation 1)

And U1 is the weighted sum of the centred dependent variable:

U_1_ = Y_0_q_1_ + E_2_ (equation 2)

The weights and the factors scores are calculated to ensure the maximum covariance between T_1_ and U_1_, which is a departure from regular PCA where the scores and loadings are calculated to explain the maximum variance in X_0_.

*So U_1_ ~ T_1_* (equation 3)

Or,

*U_1_ = B_0_ + B_1_T_1_ + E_4_* (equation 4)

Or,

*U_1_ = B_0_ + B_1_(X_0_w_1_) + E_5_* (equation 5)

In the SIMPLS algorithm provides an alternative where the matrices are not deflated by the weights when calculating the new components, and, as a result, it is easier to interpret the components based on the original centred matrices.

As the components are calculated to explain the maximum covariance between the dependent and independent variable, the first component need not explain the maximum variance in the dependent variable. However, as the number of components calculated increases, they progressively tend to explain lesser variance in the dependent variable.

*Here we present the rationale for choosing genes with both positive and negative weights:*

From equations 2 and 5 above, we know that:

*Y_0_q_1_ =* *B_0_ + B_1_(X_0_w_1_) + E_5_* (equation 6)

This can be rewritten as:

*Y_0_q_1_ ~ B_1_(X_0_w_1_)* (equation 7)

And if both B_1_ and q_1_ are positive which is the case in our analyses, then,

*Y_0_ ~ X_0_w_1_* (equation 8)

In our dataset:

Y_0_ is a p x 1 vector of ΔCT with positive and negative values.

q_1_ is a 1 x 1 vector of weight for the first PLSR component.

B_1_ is the regression coefficient.

X_0_ is a p x n matrix of gene expression, where p is the number of cortical regions, and n is the number of genes for which gene expression is calculated. This has been scaled and normalized to have positive and negative values. Positive values indicate that the gene is overexpressed compared to the mean gene expression, and negative values indicate that the gene is under-expressed compared to the mean gene expression.

w_1_ is a nx1 vector of weights for the first PLSR component.

Y_0_ can be both positive or negative (ΔCT is both positive or negative, as some regions are thicker in individuals with autism compared to controls and vice versa). Similarly, both X_0_ and w_1_ are positive or negative.

This gives us the following possibilities:

1. For a negative value in Y_0_, either the equivalent X_0_ value or the equivalent w_1_ value must be negative.
2. For a positive value in Y_0_, both the equivalent values in X_0_ and w_1_ must be either positive or negative.

In other words, if the weight of the gene is positive, having a higher-than-average gene expression (positive X_0_) contributes to positive ΔCT (i.e. greater CT in autism compared to controls), whereas having a lower than average gene expression (negative X_0_) contributes to negative ΔCT. Similarly, if the weight of the gene is negative, having a higher-than-average gene expression contributes to negative ΔCT, whereas having a lower-than-average gene expression contributes to positive ΔCT. So, the sign of the weights alone cannot tell us if the gene contributes to thicker or thinner cortex in autism compared to controls. It is the combination of both the weights and the gene expression level that can be informative. However, as gene expression and ΔCT varies considerably across the regions tested, we used genes with both positive and negative weights, that were significant FDR correction in our enrichment analyses.

### Discovery dataset

Table S2 below lists the relative information of the full 35-component model and Figure S1 shows the amount of explained variance for each component included in the final analysis. Only the first component showed a significant effect.

##### Table S2: The relative information of the full 35-component model

| **Component** | **RSS** | **RMSE** |
| --- | --- | --- |
| **1** | 14357 | 0.94576 |
| **2** | 13998 | 0.86392 |
| **3** | 18678 | 0.7759 |
| **4** | 7511.2 | 0.63904 |
| **5** | 7098.9 | 0.57656 |
| **6** | 5452.2 | 0.51071 |
| **7** | 4545.2 | 0.45452 |
| **8** | 6040.5 | 0.40239 |
| **9** | 1871.6 | 0.31855 |
| **10** | 2574.4 | 0.29152 |
| **11** | 2128.7 | 0.24715 |
| **12** | 1408.4 | 0.20464 |
| **13** | 1559.9 | 0.17398 |
| **14** | 807.52 | 0.13062 |
| **15** | 680.32 | 0.10856 |
| **16** | 540.02 | 0.088602 |
| **17** | 476.12 | 0.073443 |
| **18** | 422.29 | 0.060093 |
| **19** | 377.71 | 0.048947 |
| **20** | 359.98 | 0.040626 |
| **21** | 355.89 | 0.033044 |
| **22** | 328.2 | 0.024011 |
| **23** | 320.74 | 0.01879 |
| **24** | 315.86 | 0.014429 |
| **25** | 312.19 | 0.010714 |
| **26** | 309.59 | 0.007748 |
| **27** | 308.16 | 0.005718 |
| **28** | 307.92 | 0.004529 |
| **29** | 307.51 | 0.003291 |
| **30** | 307.27 | 0.002327 |
| **31** | 307.13 | 0.001597 |
| **32** | 307.06 | 0.001063 |
| **33** | 307.02 | 0.000715 |
| **34** | 307.01 | 0.000517 |
| **35** | 307.01 | 0.000369 |

*For each component the Residual Sum of Squares (RSS) and the Root Mean Square of the Error (RMSE) are provided.*

#####
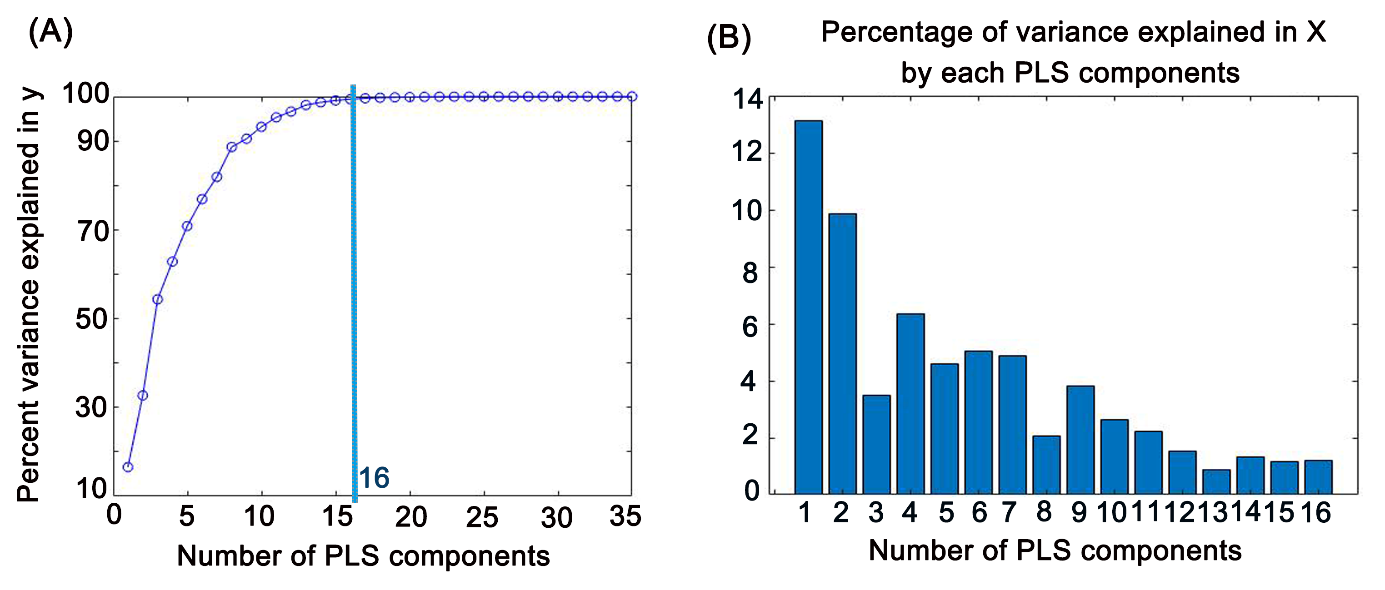
Figure S1: Variance in X (gene expression) explained by PLS components

*The cross-validation analysis identified that 16 components provide the best model fit (Figure S1(A)). Only components 1 and 2 explained more than 10% of the total variance* *respectively, and were thus selected for further analyses. Of these 2, only component 1 explained a significant proportion of the variance in ΔCT.* *Thus, we selected PLSR component 1 (PLSR1) for next analyses.*

##### Table S3: The main enrichment result for PLSR1 in Discovery dataset (MCI_S VS. MCI-AD)

| **Term** | **Description** | **Log10P** | **Log10(q)** | **overlap** |
| --- | --- | --- | --- | --- |
| GO:0007268 | **chemical synaptic transmission** | -24.13037079 | -20.072 | 100/684 |
| GO:0007268 | chemical synaptic transmission | -24.13037079 | -20.072 | 100/684 |
| GO:0098916 | anterograde trans-synaptic signaling | -24.13037079 | -20.072 | 100/684 |
| GO:0099536 | synaptic signaling | -23.92137578 | -20.040 | 101/700 |
| GO:0099537 | trans-synaptic signaling | -23.78633663 | -20.029 | 100/691 |
| GO:0050804 | modulation of chemical synaptic transmission | -14.27800663 | -11.065 | 60/418 |
| GO:0099177 | regulation of trans-synaptic signaling | -14.23022892 | -11.047 | 60/419 |
| GO:0007611 | learning or memory | -9.624026 | -6.784 | 36/235 |
| GO:0007610 | behavior | -9.38591045 | -6.558 | 60/544 |
| GO:0050890 | cognition | -8.761136913 | -5.970 | 38/276 |
| GO:0007612 | learning | -5.675670526 | -3.417 | 20/130 |
| GO:0032990 | cell part morphogenesis | -20.43496771 | -16.775 | 93/679 |
| R-HSA-112316 | Neuronal System | -17.62969895 | -14.271 | 65/410 |
| GO:0007420 | brain development | -13.71483169 | -10.560 | 82/710 |
| GO:0099504 | synaptic vesicle cycle | -12.48276885 | -9.403 | 35/179 |
| GO:0050808 | synapse organization | -11.87715103 | -8.819 | 55/411 |
| GO:0021953 | central nervous system neuron differentiation | -10.32840245 | -7.384 | 32/180 |
| R-HSA-794362 | Protein-protein interactions at synapses | -8.800044706 | -5.997 | 20/86 |
| GO:0042391 | regulation of membrane potential | -8.658494086 | -5.902 | 50/431 |

### *Note: The shade of blue color represents the size of the Log10 p value*

### Validation dataset 1

For the validation dataset 1, we conducted the same analysis as that in discovery dataset.

##### Figure S2: Variance in X (gene expression) explained by PLS components


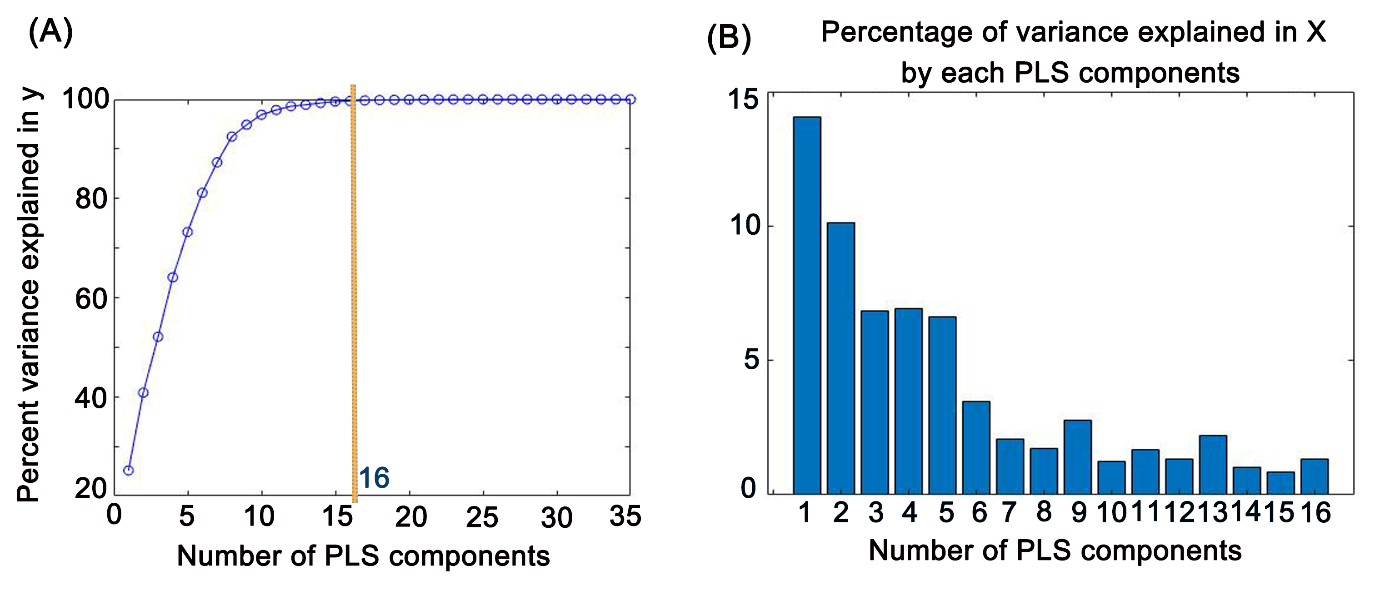


*The cross-validation analysis identified that 16 components provide the best model fit (Figure S2(A)). Only components 1 and 2 explained more than 10% of the total variance respectively, and were thus selected for further analyses. Of these 2, only component 1 explained a significant proportion of the variance in ΔCT (Figure S2(B)).*

##### Table S4: The main enrichment result for PLSR1 in validation dataset (NC VS. MCI_S)

| **Term** | **Description** | **Log10P** | **Log10(q)** | **overlap** |
| --- | --- | --- | --- | --- |
| GO:0007268 | **chemical synaptic transmission** | -23.66857388 | -19.743 | 159/684 |
| GO:0098916 | anterograde trans-synaptic signaling | -23.66857388 | -19.743 | 159/684 |
| GO:0099537 | trans-synaptic signaling | -23.62526315 | -19.743 | 160/691 |
| GO:0099536 | synaptic signaling | -23.44408439 | -19.687 | 161/700 |
| GO:0099177 | regulation of trans-synaptic signaling | -14.08248202 | -11.025 | 96/419 |
| GO:0050804 | modulation of chemical synaptic transmission | -13.71389326 | -10.677 | 95/418 |
| GO:0050806 | positive regulation of synaptic transmission | -5.664469398 | -3.812 | 36/158 |
| GO:0048167 | regulation of synaptic plasticity | -3.579007801 | -2.147 | 33/174 |
| GO:0060291 | long-term synaptic potentiation | -2.882280395 | -1.623 | 18/83 |
| GO:0043269 | regulation of ion transport | -17.01539768 | -13.355 | 145/696 |
| GO:0042391 | regulation of membrane potential | -16.85083461 | -13.270 | 104/431 |
| GO:0060322 | head development | -16.6252244 | -13.154 | 152/751 |
| GO:0050808 | synapse organization | -16.01681612 | -12.612 | 99/411 |
| GO:0009636 | response to toxic substance | -15.60683431 | -12.248 | 113/506 |
| GO:1905114 | cell-cell signaling | -15.49400827 | -12.176 | 129/614 |
| R-HSA-675108 | Nervous system development | -14.95801522 | -11.678 | 122/576 |
| hsa04080 | Neuroactive ligand-receptor interaction | -14.66875359 | -11.424 | 90/373 |
| GO:0032990 | cell part morphogenesis | -14.55077061 | -11.338 | 136/679 |

### Specificity dataset 1

For the specificity dataset 1, we conducted the same PLSR analysis as that in discovery dataset. Figure S3 shows the result. However, enrichment analysis of the first PLSR component was significantly associated with the GO term “*detection of stimulus involved in sensory perception*” (Figure S4) and not enrichment in GO term “*Chemical synaptic transmission*”. This result well verifies that the conclusion of our study was MCI specific (degeneration from MCI to AD). We discussed it in the main text.

##### Figure S3: Variance in X (gene expression) explained by PLS components


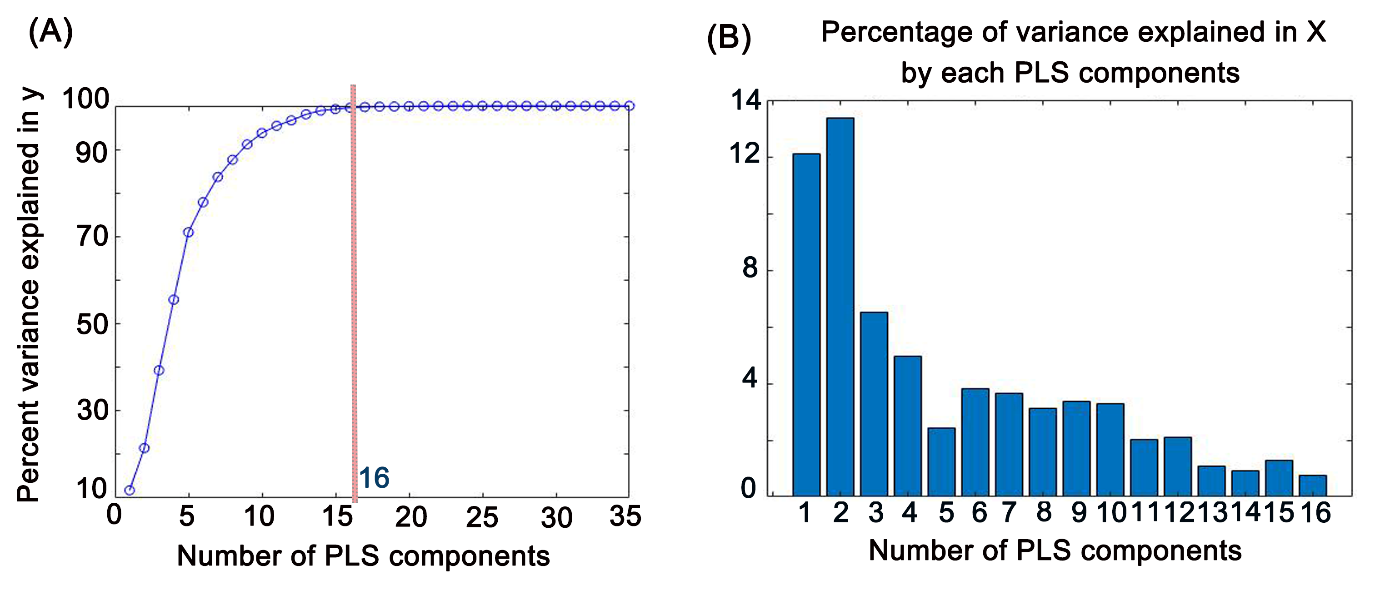


*The cross-validation analysis identified that 16 components provide the best model fit Figure S2(A). Only components 1 and 2 explained more than 10% of the total variance respectively, and were thus selected for further analyses. Of these 2, only component 1 explained a significant proportion of the variance in ΔCT.*

##### Figure S4: Enrichment analysis result of Specificity dataset 1.


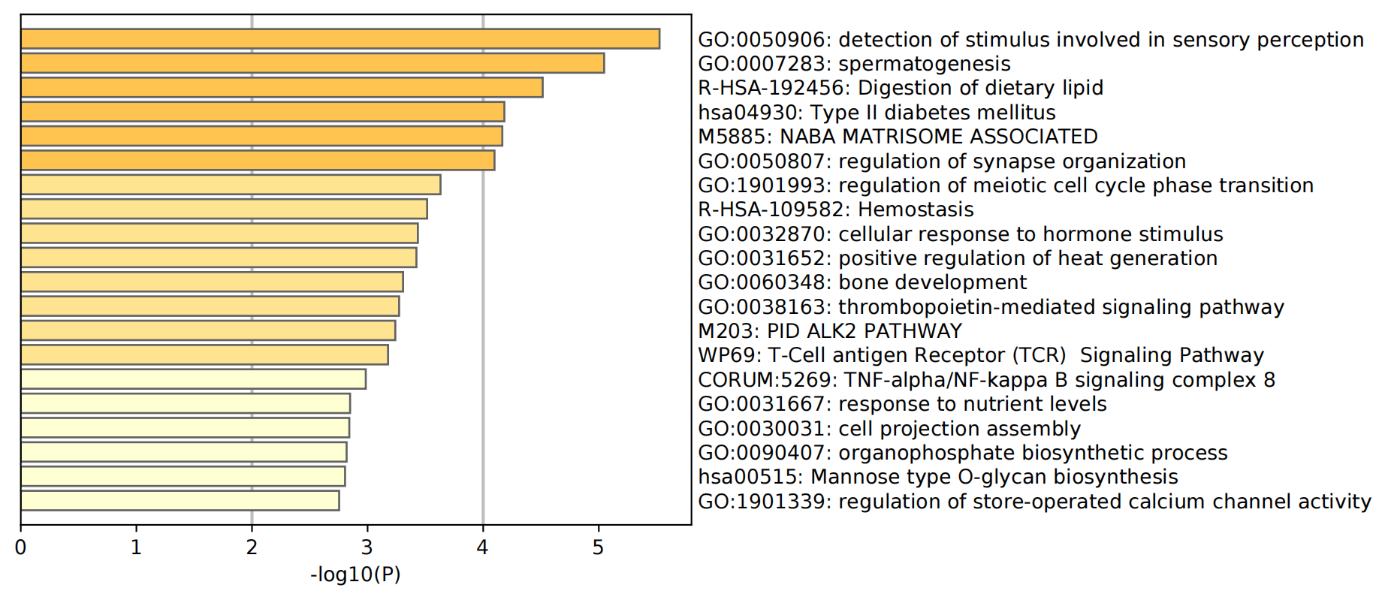


## 4. WGCNA Analysis

To verify that our results was not due to the use of the PLSR method, we applied the Weighted gene co-expression network analysis (WGCNA) method to verify the robustness of our results. WGCNA is a systems biology method for describing the correlation patterns among genes across microarray samples ([Langfelder and Horvath, 2008](#_ENREF_9)). WGCNA not only focus on differentially expressed genes but also combine genes with phenotypes. The common between PSLR and WGCNA is that those could establish the associations between transcriptional expression of genes and phenotypic characteristics, acting as a bridge. The difference between the two methods is that those use different algorithms. The algorithm and data processing procedures of PLSR are mentioned above. WGCNA can be used for finding clusters (modules) of highly correlated genes, for summarizing such clusters using the module eigengene (each module gene and sample matrix were analyzed by PCA for the first principal component) or an intramodular hub gene, for relating modules to one another and to external sample traits (using eigengene network methodology), and for calculating module membership measures (2-3). Correlation networks facilitate network based gene screening methods that can be used to identify candidate biomarkers or therapeutic targets. These methods have been successfully applied in various biological contexts, e.g. cancer, mouse genetics, and analysis of brain imaging data (4-6). More detailed algorithms are available in Langfelder P et al., (2008)(2). Although there are algorithmic differences between the two methods, [all](D:/youdao/Dict/8.9.9.0/resultui/html/index.html#/javascript:;) [roads](D:/youdao/Dict/8.9.9.0/resultui/html/index.html#/javascript:;) [lead](D:/youdao/Dict/8.9.9.0/resultui/html/index.html#/javascript:;) [to](D:/youdao/Dict/8.9.9.0/resultui/html/index.html#/javascript:;) [Rome](D:/youdao/Dict/8.9.9.0/resultui/html/index.html#/javascript:;). The goal of our work is to detect enriched pathways of genes associated with cortical thickness differences (ΔCT). [To](D:/youdao/Dict/8.9.9.0/resultui/html/index.html#/javascript:;) [a](D:/youdao/Dict/8.9.9.0/resultui/html/index.html#/javascript:;) [large](D:/youdao/Dict/8.9.9.0/resultui/html/index.html#/javascript:;) [extent](D:/youdao/Dict/8.9.9.0/resultui/html/index.html#/javascript:;), It is rational to apply WGCNA to verify the results of PSLR.

We applied this method to explore the relationship between *ΔCT* and gene expression of from AIBS (the same gene expression dataset in PLSR). It’s an approach that select co-expression gene modules and construct association between modules and *ΔCT*. Ultimately, the highly co-expressed gene modules were divided into different modules with different colors (Figure S5). We used the R package to visualization of the module results. In the end, we extracted genes from the most correlation modules (top 2, MElightcyan and MEtan, Figure S6) to perform the enrichment analysis. Enrichment result is shown in Table S4 and Figure 4 in main text.

### Discovery dataset and validation dataset 1

The two groups were analyzed together because they used the WGCNA method.

##### Figure S5: co-expressed gene modules were divided into different modules with different colours.


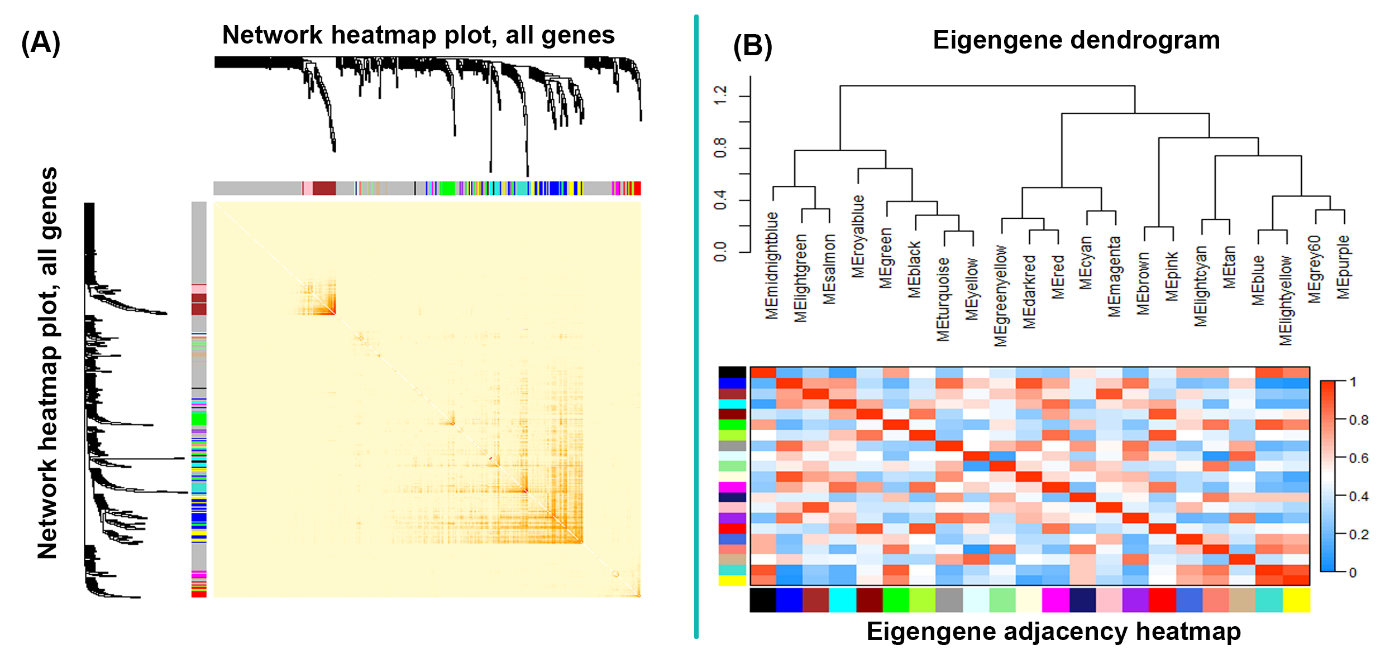


#####
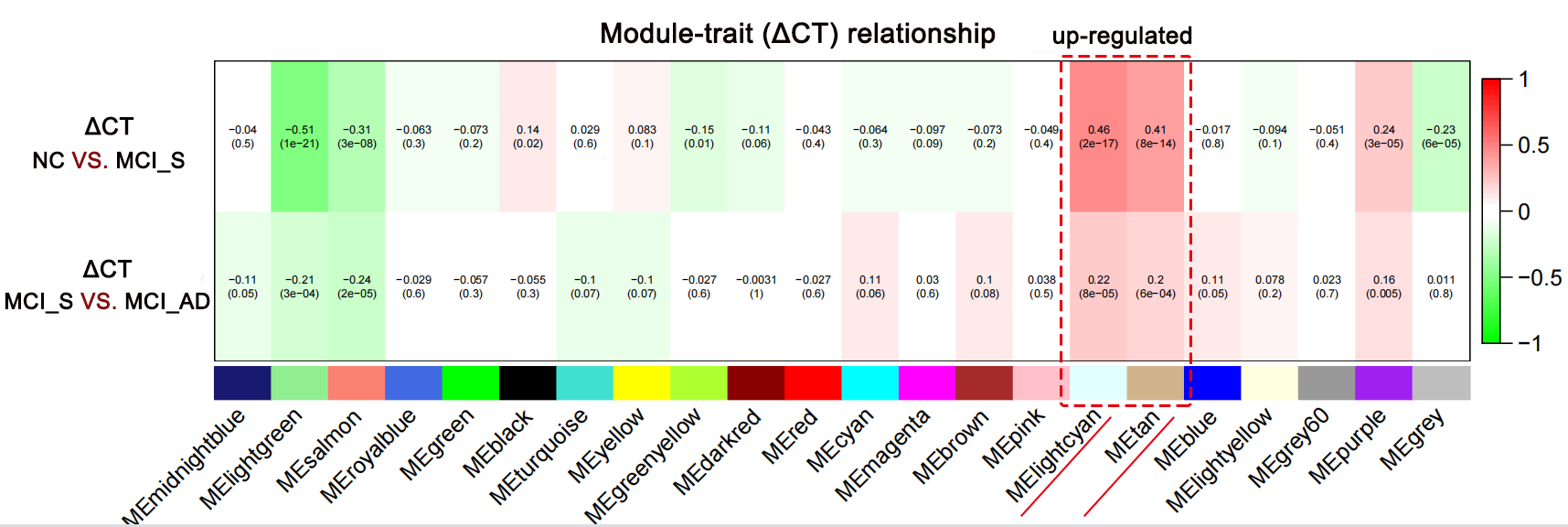
Figure S6: Module-trait (ΔCT) relationship in NC VS. MCI_S and MCI_S VS.MCI_AD.

##### Table S5: The main enrichment result using WGCNA in discovery and validation datasets

| **Term** | **Description** | **Log10P** | **Log10(q)** | **Overlap** |
| --- | --- | --- | --- | --- |
| GO:0007268 | **chemical synaptic transmission** | -18.99839889 | -14.963 | 39/686 |
| GO:0023061 | signal release | -9.104748524 | -5.590 | 22/477 |
| R-HSA-72790 | Signaling by GPCR | -8.61518133 | -5.159 | 26/704 |
| R-HSA-00792 | GPCR ligand binding | -7.783233727 | -4.401 | 20/467 |
| R-HSA-88396 | GPCR downstream signaling | -7.594082215 | -4.276 | 23/629 |
| GO:0035249 | synaptic transmission, glutamatergic | -6.806304564 | -3.561 | 9/90 |
| GO:0051966 | regulation of synaptic transmission | -5.456812346 | -2.573 | 7/69 |
| GO:0050890 | cognition | -6.483312205 | -3.285 | 14/276 |
| GO:0050433 | regulation of catecholamine secretion | -6.305414665 | -3.150 | 7/52 |
| GO:0050432 | catecholamine secretion | -6.247309599 | -3.118 | 7/53 |
| GO:0015844 | monoamine transport | -6.153552496 | -3.099 | 8/79 |
| GO:0007611 | learning or memory | -5.667726289 | -2.706 | 12/235 |
| GO:0007610 | behavior | -5.455596997 | -2.573 | 18/543 |
| GO:0050808 | synapse organization | -6.468805529 | -3.285 | 17/412 |
| GO:0006836 | neurotransmitter transport | -6.180712815 | -3.100 | 12/210 |
| GO:0060627 | regulation of vesicle-mediated transport | -5.741068585 | -2.762 | 18/518 |

### Specificity dataset 1

For the specificity dataset 1, we also conducted the WGCNA analysis. The highly co-expressed gene modules with different colors are similar with Figure S5 (because we use the same parameters with the discovery dataset and validation dataset 1). We used the R package to merge the module results of the three datasets, which is convenient to compare the differences and similarities between the results of the three datasets (Figure S7). In the end, we extracted genes from the most correlation modules (MElightgreen, Figure S7) to perform the enrichment analysis. Genes of the MElightgreen module was not enriched in GO term “*Chemical synaptic transmission*” (Figure S8).

##### Figure S7: Module-trait (ΔCT) relationship in discovery dataset, validation dataset 1 and specificity dataset 1.


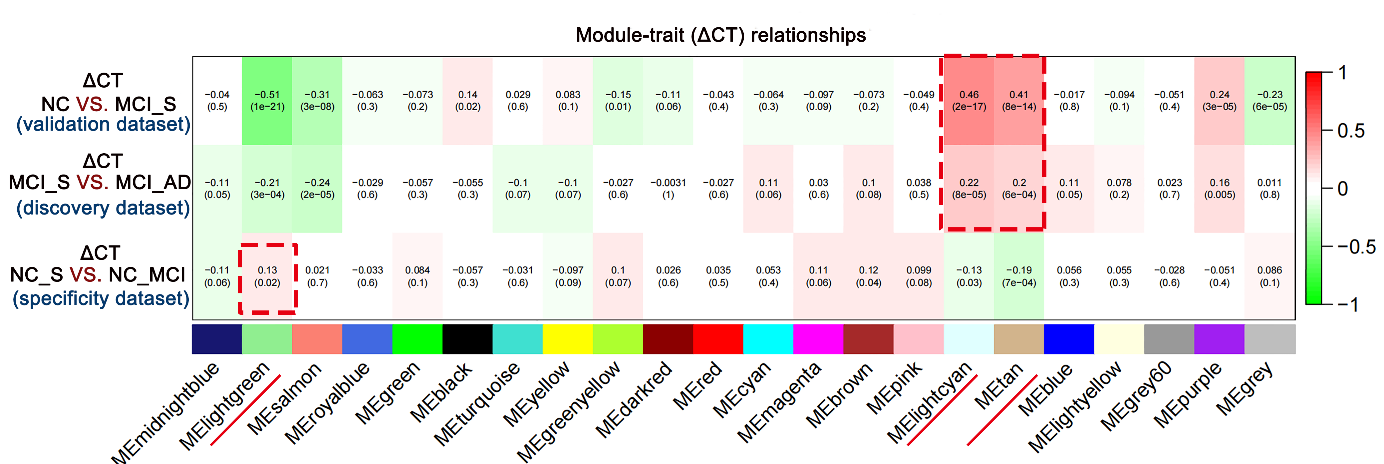


##### Figure S8: Enrichment analysis result in specificity dataset 1.


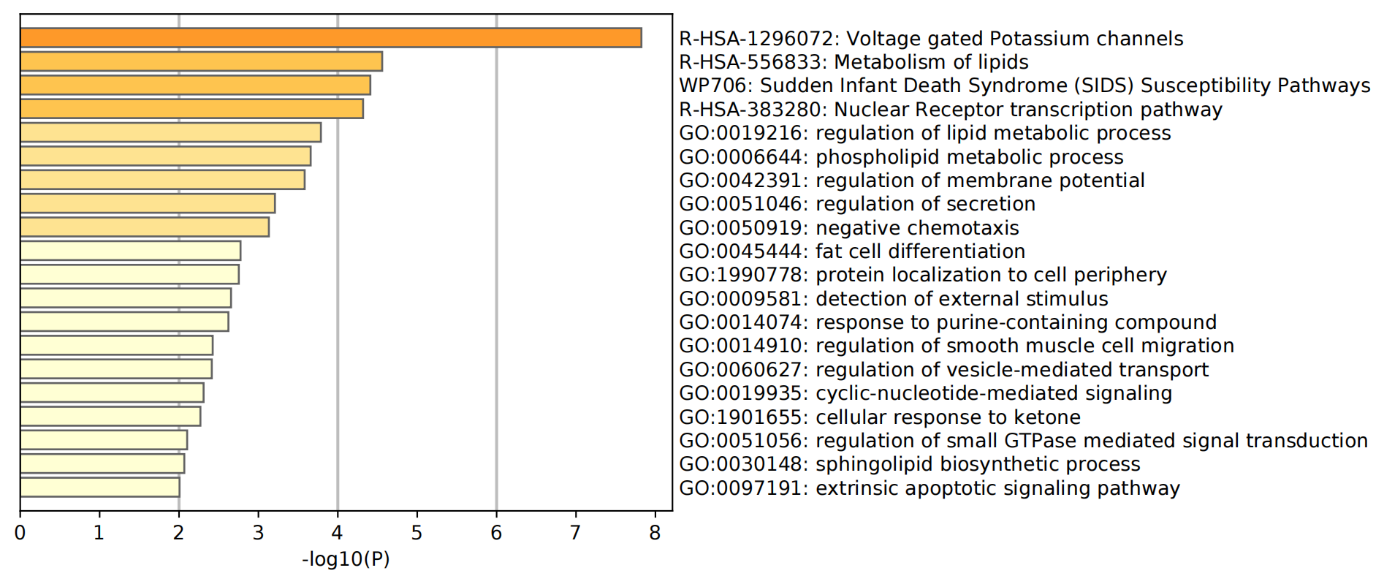


## 5. Indirect Validation and Specificity Analysis

Tissue-specific enrichment analysis shows that the prefrontal lobe is the coincident brain tissue both in the discovery and validation datasets (Table S6). Thus, we conducted validation and specificity analyses purely from the perspective of gene expression from prefrontal lobe from GEO database to indirectly verify our results.

##### Table S6: Tissue-specific enrichment analysis

| Discovery dataset (MCI_S VS. MCI_AD); PLSR analysis | | | | | |
| --- | --- | --- | --- | --- | --- |
| Term | Overlap | P-value | Adjusted P-value | Odds Ratio | Combined Score |
| Amygdala | 72/345 | 8.43E-18 | 6.91E-16 | 3.736060236 | 146.8836684 |
| Fetal brain | 55/325 | 3.68E-10 | 1.51E-08 | 2.848748977 | 61.87981143 |
| Prefrontal Cortex | 56/521 | 5.26E-04 | 0.014378299 | 1.667571466 | 12.5904017 |
| Validation dataset 1 (NC VS. MCI_S); PLSR analysis | | | | | |
| Term | Overlap | P-value | Adjusted P-value | Odds Ratio | Combined Score |
| Prefrontal Cortex | 68/521 | 1.45E-06 | 1.17E-04 | 1.973346415 | 26.53345217 |
| pineal night | 77/674 | 4.13E-05 | 0.001671456 | 1.693122043 | 17.09267974 |
| Cerebellum | 19/108 | 2.50E-04 | 0.00674307 | 2.764501946 | 22.93175616 |
| Thalamus | 14/79 | 0.001453 | 0.029437641 | 2.782991991 | 18.18305641 |

### Validation dataset 2 (GEO)

Genetic expression data of the brain prefrontal cortex is from GEO database. Limma package (version: 3.40.2) of R software was used to study the differential expression. The adjusted P-value was analyzed to correct for false positive results in GEO datasets. “A_djusted_ P < 0.01 and Log (Fold Change) >2 or Log (Fold Change) < −2” were defined as the thresholds for the screening of differential expression. Then, the data were analyzed by functional enrichment (Gene Ontology (GO) and Kyoto Encyclopedia of Genes and Genomes (KEGG)) in metascape tool (http://metascape.org/) ([Yingyao et al., 2019](#_ENREF_15)). ClusterProfiler package (version: 3.18.0) in R was employed to analyze the GO function of potential targets and enrich the KEGG pathway. The box plot is implemented by the R software package ggplot2; PCA graphs are drawn by R software package ggord; the heat map is displayed by the R software package pheatmap. Detailed information in Figure S9. All the above analysis methods and R package were implemented by R foundation for statistical computing (2020) version 4.0.3. Enrichment analysis was also significantly associated with the GO term “Chemical synaptic transmission” (Table S7).

#####
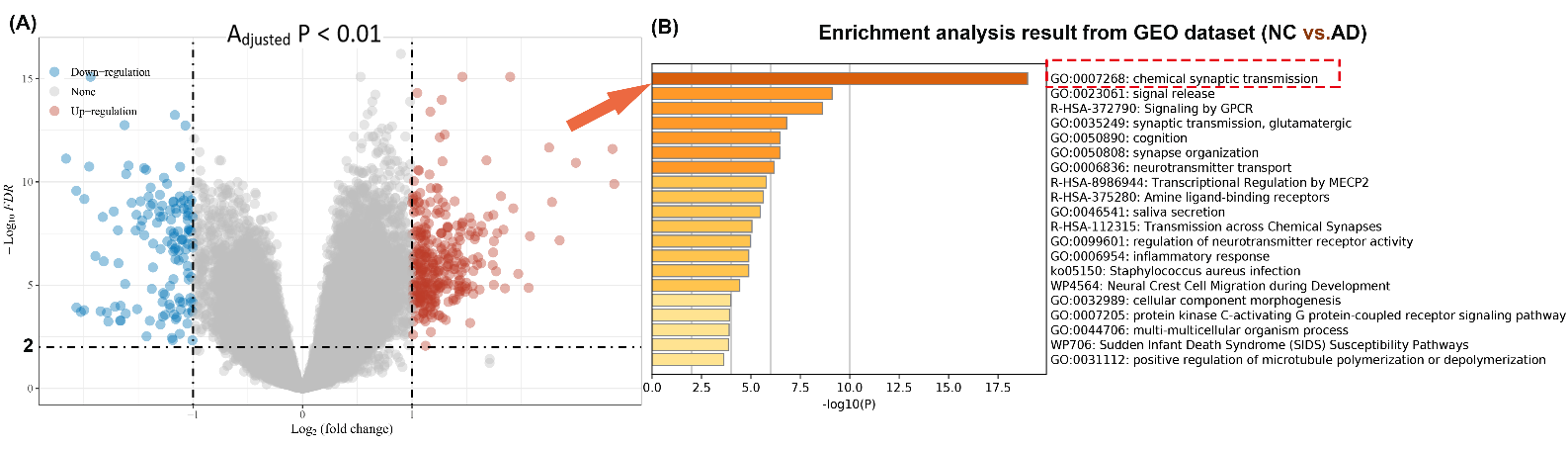
Figure S9: Differential expression analysis and enrichment analysis results

##### Table S7: The main enrichment result in validation dataset 2 (GEO, NC VS. AD)

| **Term** | **Description** | **Log10P** | **Log10(q)** | **Overlap** |
| --- | --- | --- | --- | --- |
| GO:0007268 | **chemical synaptic transmission** | -18.99839889 | -14.963 | 39/686 |
| GO:0098916 | anterograde trans-synaptic signaling | -18.99839889 | -14.963 | 39/686 |
| GO:0099537 | trans-synaptic signaling | -18.845355 | -14.963 | 39/693 |
| GO:0099536 | synaptic signaling | -18.37612303 | -14.619 | 39/715 |
| GO:0050804 | modulation of chemical synaptic transmission | -9.628002441 | -6.027 | 21/405 |
| GO:0099177 | regulation of trans-synaptic signaling | -9.608491856 | -6.027 | 21/406 |
| GO:0050806 | positive regulation of synaptic transmission | -9.104748524 | -2.991 | 10/144 |
| GO:0023061 | signal release | -8.61518133 | -5.590 | 22/477 |
| R-HSA-32790 | Signaling by GPCR | -8.61518133 | -5.159 | 26/704 |
| R-HSA-500792 | GPCR ligand binding | -7.594082215 | -4.401 | 20/467 |
| R-HSA-388396 | GPCR downstream signalling | -6.806304564 | -4.276 | 23/629 |
| ko04080 | Neuroactive ligand-receptor interaction | -6.806304564 | -2.193 | 12/277 |
| R-HSA-416476 | G alpha (q) signalling events | -6.483312205 | -1.840 | 10/216 |
| GO:0035249 | synaptic transmission, glutamatergic | -6.483312205 | -3.561 | 9/90 |
| GO:0035249 | synaptic transmission, glutamatergic | -6.468805529 | -3.561 | 9/90 |
| GO:0051966 | regulation of synaptic transmission | -6.468805529 | -2.573 | 7/69 |
| GO:0050890 | cognition | -6.305414665 | -3.285 | 14/276 |
| GO:0050433 | regulation of catecholamine secretion | -6.180712815 | -3.150 | 7/52 |
| GO:0050432 | catecholamine secretion | -6.180712815 | -3.118 | 7/53 |
| GO:0015844 | monoamine transport | -6.153552496 | -3.099 | 8/79 |
| GO:0007611 | learning or memory | -6.007732715 | -2.706 | 12/235 |
| GO:0051937 | catecholamine transport | -5.774149195 | -2.573 | 7/68 |
| GO:0007610 | behavior | -5.774149195 | -2.573 | 18/543 |

### Specificity dataset 2 (GEO)

For the specificity dataset 2, we conducted the same analysis on genetic expression data from the brain prefrontal cort from GEO database in sample of VaD and normal controls. “A_djusted_ P < 0.01 and Log (Fold Change) >2 or Log (Fold Change) < −2” were defined as the thresholds for the screening of differential expression of mRNAs. However, there were no differentially expressed genes. “A_djusted_ P < 0.05 and Log (Fold Change) >1 or Log (Fold Change) < −1” were defined as the thresholds for the screening of differential expression of mRNAs. There were almost no differentially expressed genes (2 up-regulated genes and 2 down-regulated genes) (Figure S10). Therefore, we did not carry out the enrichment analysis in the next step. This result still well verifies that the conclusion of our study was MCI specific.

#####
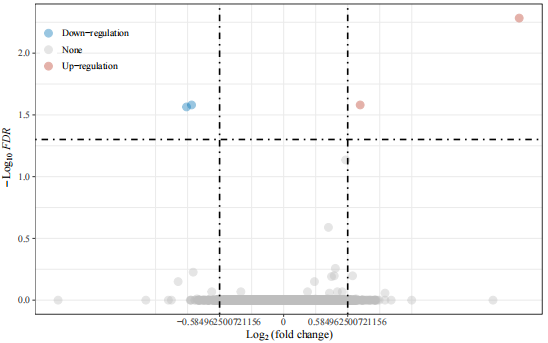
Figure S10: Differential expression analysis in sample of VaD and normal controls

## 6. KEGG based pathway enrichment

We also conducted KEGG based pathway enrichment for the discovery and validation datasets using Metascape tool (http://metascape.org/) ([Yingyao et al., 2019](#_ENREF_15)). Top 2-10 pathways are provided as follows (Tables S8). The main KEGG pathways are “Calcium signaling pathway”, “Glutamatergic synapse”,” Cholinergic synapse” and “Neuroactive ligand-receptor interaction”.

##### Table S8: Top 2-10 pathways enrichment for the discovery and validation datasets using PLSR and WGCNA respectively.

| Top 10 pathways (Discovery dataset (MCI_S VS. MCI_AD); PLSR analysis) | | | |
| --- | --- | --- | --- |
| **Term** | **Description** | **Log10 P** | **Log(q)** |
| hsa04360 | Axon guidance | -7.99142 | -3.578 |
| hsa04724 | Glutamatergic synapse | -7.77473 | -5.108 |
| hsa04726 | Serotonergic synapse | -7.6695 | -2.961 |
| ko04020 | Calcium signaling pathway | -7.52173 | -2.854 |
| hsa04725 | Cholinergic synapse | -6.80832 | -2.672 |
| hsa04540 | Gap junction | -5.89576 | -2.322 |
| hsa04728 | Dopaminergic synapse | -5.00556 | -1.719 |
| hsa04022 | cGMP-PKG signaling pathway | -4.32451 | -1.098 |
| ko04071 | Sphingolipid signaling pathway | -4.28266 | -1.066 |
| ko04080 | Neuroactive ligand-receptor interaction | -2.47969 | -5.027 |
| Top 2 pathways (Validation dataset 1 (NC VS. MCI_S); PLSR analysis) | | | |
| **Term** | **Description** | **Log10 P** | **Log(q)** |
| hsa04080 | Neuroactive ligand-receptor interaction | -14.66875359 | -11.424 |
| ko04360 | Axon guidance | -5.40545 | -3.595 |
| Top 10 pathways (Validation datasets (MCI_S VS.MCI_AD; NC VS.MCI_S); WGCNA analysis) | | | |
| **Term** | **Description** | **Log10 P** | **Log(q)** |
| hsa04080 | Neuroactive ligand-receptor interaction | -12.5858 | -8.722 |
| hsa04024 | cAMP signaling pathway | -7.0994 | -4.019 |
| ko04723 | Retrograde endocannabinoid signaling | -6.50852 | -3.547 |
| ko04724 | Glutamatergic synapse | -5.09486 | -2.389 |
| ko04727 | GABAergic synapse | -5.04098 | -2.355 |
| hsa04750 | inflammatory mediator regulation of trp channels | -3.64848 | -1.375 |
| hsa04062 | Chemokine signaling pathway | -3.51299 | -1.312 |
| hsa04725 | Cholinergic synapse | -3.38142 | -1.215 |
| hsa04371 | Apelin signaling pathway | -2.77132 | -0.810 |
| hsa04728 | Dopaminergic synapse | -2.33257 | -0.543 |
| Top 4 pathways (Validation dataset2 (GEO; NC-AD)) | | | |
| **Term** | **Description** | **Log10 P** | **Log(q)** |
| ko04080 | Neuroactive ligand-receptor interaction | -12.5958 | -9.705 |
| hsa04725 | Cholinergic synapse | -4.92212 | -2.193 |
| hsa04810 | Regulation of actin cytoskeleton | -3.07923 | -0.927 |
| hsa04020 | Calcium signaling pathway | -2.15186 | -0.289 |

## 7. Enrichment analysis in DisGeNET

We also conducted enrichment analysis in DisGeNET ([Piñero et al., 2016](#_ENREF_11)) for the intersection genes of discovery and validation dataset using Metascape tool (http://metascape.org/) ([Yingyao et al., 2019](#_ENREF_15)). The intersection genes of the two datasets were 94. Those mainly enriched in cognition disorders, amyloidosis, Alzheimer’s disease, and memory impairment. Details are provided in Figure S11.

##### Figure S11: Enrichment analysis result in DisGeNET


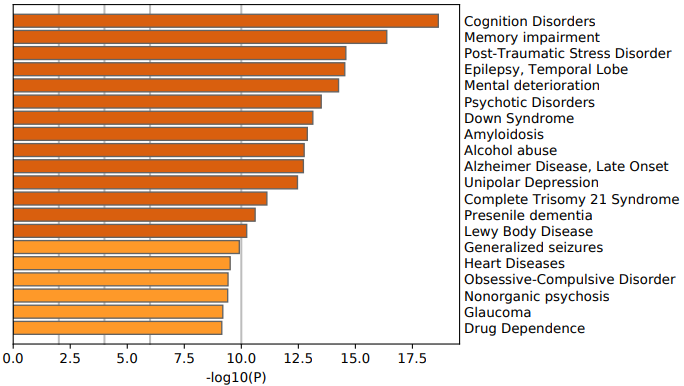


## 8. Protein-protein interaction enrichment analysis

For each given gene list, protein-protein interaction enrichment analysis has been carried out with the following databases: STRING ([Szklarczyk et al., 2019](#_ENREF_14)) BioGrid (Stark C. et al. 2006), InWeb_IM and OmniPath ([Li et al., 2017](#_ENREF_10)). Only physical interactions in STRING (physical score > 0.132) and BioGrid are used. The resultant network contains the subset of proteins that form physical interactions with at least one other member in the list. If the network contains between 3 and 500 proteins, the Molecular Complex Detection (MCODE) algorithm has been applied to identify densely connected network components ([Bader and Hogue, 2003](#_ENREF_2)).

## 9. Von Economo classification

Previous researches recorded an association between cortical thickness and cytoarchitectural cortical features linked to specific abnormalities in laminar thickness of supragranular layers of the cortex of MCI or AD patients. Thus, we conducted spatial expression profiling of selected PLSR component across all 5 Von Economo classes and the additional 2 subtype classes covering limbic regions and allocortex (class 6) and insular cortex (class 7). The 7 classes are Class 1: granular cortex, primary motor cortex. Class 2: association cortex. Class 3: association cortex. Class 4: dysgranular cortex, secondary sensory cortex. Class 5: agranular cortex, primary sensory cortex. Class 6: limbic regions, allocortex. Class 7: insular cortex ([Economo et al., 2008](#_ENREF_4)).

## References

Anderson, K.M., Krienen, F.M., Choi, E.Y., Reinen, J.M., Yeo, B.T., Holmes, A.J., 2018. Gene expression links functional networks across cortex and striatum. Nature communications 9, 1-14.

Bader, G.D., Hogue, C.W., 2003. An automated method for finding molecular complexes in large protein interaction networks. BMC bioinformatics 4, 1-27.

Desikan, R.S., Ségonne, F., Fischl, B., Quinn, B.T., Dickerson, B.C., Blacker, D., Buckner, R.L., Dale, A.M., Maguire, R.P., Hyman, B.T., 2006. An automated labeling system for subdividing the human cerebral cortex on MRI scans into gyral based regions of interest. Neuroimage 31, 968-980.

Economo, C.V., Koskinas, G.N., Triarhou, L.C., 2008. Atlas of Cytoarchitectonics of the Adult Human Cerebral Cortex. Atlas of Cytoarchitectonics of the Adult Human Cerebral Cortex.

Fischl, B., 2012. FreeSurfer. Neuroimage 62, 774-781.

Gorgolewski, K.J., Alfaro-Almagro, F., Auer, T., Bellec, P., Capotă, M., Chakravarty, M.M., Churchill, N.W., Cohen, A.L., Craddock, R.C., Devenyi, G.A., 2017. BIDS apps: Improving ease of use, accessibility, and reproducibility of neuroimaging data analysis methods. PLoS computational biology 13, e1005209.

Hawrylycz, M., Miller, J.A., Menon, V., Feng, D., Dolbeare, T., Guillozet-Bongaarts, A.L., Jegga, A.G., Aronow, B.J., Lee, C.-K., Bernard, A., 2015. Canonical genetic signatures of the adult human brain. Nature neuroscience 18, 1832.

Hawrylycz, M.J., Lein, E.S., Guillozet-Bongaarts, A.L., Shen, E.H., Ng, L., Miller, J.A., Van De Lagemaat, L.N., Smith, K.A., Ebbert, A., Riley, Z.L., 2012. An anatomically comprehensive atlas of the adult human brain transcriptome. Nature 489, 391-399.

Langfelder, P., Horvath, S., 2008. WGCNA: an R package for weighted correlation network analysis. BMC bioinformatics 9, 559.

Li, T., Wernersson, R., Hansen, R.B., Horn, H., Mercer, J., Slodkowicz, G., Workman, C.T., Rigina, O., Rapacki, K., Stærfeldt, H.H., 2017. A scored human protein–protein interaction network to catalyze genomic interpretation. Nature methods 14, 61.

Piñero, J., Bravo, À., Queralt-Rosinach, N., Gutiérrez-Sacristán, A., Deu-Pons, J., Centeno, E., García-García, J., Sanz, F., Furlong, L.I., 2016. DisGeNET: a comprehensive platform integrating information on human disease-associated genes and variants. Nucleic acids research, gkw943.

Romero-Garcia, R., Atienza, M., Clemmensen, L.H., Cantero, J.L., 2012. Effects of network resolution on topological properties of human neocortex. Neuroimage 59, 3522-3532.

Romero-Garcia, R., Warrier, V., Bullmore, E.T., Baron-Cohen, S., Bethlehem, R.A., 2019. Synaptic and transcriptionally downregulated genes are associated with cortical thickness differences in autism. Molecular psychiatry 24, 1053-1064.

Szklarczyk, D., Gable, A.L., Lyon, D., Junge, A., Wyder, S., Huerta-Cepas, J., Simonovic, M., Doncheva, N.T., Morris, J.H., Bork, P., 2019. STRING v11: protein–protein association networks with increased coverage, supporting functional discovery in genome-wide experimental datasets. Nucleic acids research 47, D607-D613.

Yingyao, Zhou, Bin, Lars, Pache, Max, Chang, Alireza, Hadj, Khodabakhshi, 2019. Metascape provides a biologist-oriented resource for the analysis of systems-level datasets. Nature Communications.

de Jong, S. (1993). SIMPLS: An alternative approach to partial least squares regression. Chemom. Intell. Lab. Syst. 18, 251–263.
